# Remodeling lesions locate at sites of strong extravillous trophoblast invasion and are associated with neutrophil presence in the human first-trimester decidua

**DOI:** 10.1093/humrep/deag078

**Published:** 2026-06-05

**Authors:** G Moser, D Kummer, M Gruber, T Hartmann, D Forstner, J Guettler, M Siwetz, M Sundl, C Daxboeck, S Kienesberger, J Kargl, A Deutsch, J Fessler, B Huppertz, J Feichtinger, M Gauster

**Affiliations:** Division of Cell Biology, Histology and Embryology, Gottfried Schatz Research Center, Medical University of Graz, Graz, Austria; BioTechMed-Graz, Graz, Austria; Division of Cell Biology, Histology and Embryology, Gottfried Schatz Research Center, Medical University of Graz, Graz, Austria; Division of Cell Biology, Histology and Embryology, Gottfried Schatz Research Center, Medical University of Graz, Graz, Austria; Division of Cell Biology, Histology and Embryology, Gottfried Schatz Research Center, Medical University of Graz, Graz, Austria; Division of Cell Biology, Histology and Embryology, Gottfried Schatz Research Center, Medical University of Graz, Graz, Austria; Division of Cell Biology, Histology and Embryology, Gottfried Schatz Research Center, Medical University of Graz, Graz, Austria; Department of Obstetrics and Gynaecology, NIHR Cambridge Biomedical Research Centre, University of Cambridge, Cambridge, UK; The Loke Centre for Trophoblast Research, Department of Physiology, Development and Neuroscience; University of Cambridge, Cambridge, UK; Division of Cell Biology, Histology and Embryology, Gottfried Schatz Research Center, Medical University of Graz, Graz, Austria; Division of Cell Biology, Histology and Embryology, Gottfried Schatz Research Center, Medical University of Graz, Graz, Austria; Division of Cell Biology, Histology and Embryology, Gottfried Schatz Research Center, Medical University of Graz, Graz, Austria; BioTechMed-Graz, Graz, Austria; Institute of Molecular Biosciences, University of Graz, Graz, Austria; Field of Excellence BioHealth, University of Graz, Graz, Austria; BioTechMed-Graz, Graz, Austria; Division of Pharmacology, Otto Loewi Research Institute, Medical University of Graz, Graz, Austria; Division of Hematology, Medical University of Graz, Graz, Austria; Division of Immunology, Otto Loewi Research Center, Medical University of Graz, Graz, Austria; Division of Cell Biology, Histology and Embryology, Gottfried Schatz Research Center, Medical University of Graz, Graz, Austria; Division of Cell Biology, Histology and Embryology, Gottfried Schatz Research Center, Medical University of Graz, Graz, Austria; BioTechMed-Graz, Graz, Austria; Division of Cell Biology, Histology and Embryology, Gottfried Schatz Research Center, Medical University of Graz, Graz, Austria

**Keywords:** first-trimester decidua, placental development, extravillous trophoblast, EVTs, EVT invasion, trophoblast invasion, immune microenvironment, remodeling lesion, placental anatomy, spatial transcriptomics

## Abstract

**STUDY QUESTION:**

Does the degree of extravillous trophoblast (EVT) invasion influence decidual tissue architecture and immune cell distribution in first-trimester decidua?

**SUMMARY ANSWER:**

Areas of pronounced morphological changes have been identified at sites of strong EVT invasion in the *decidua basalis*—defined as ‘remodeling lesions’—and are associated with a substantially reshaped immune cell landscape.

**WHAT IS KNOWN ALREADY:**

During early human placental development, EVTs invade the decidua to facilitate placental attachment and nutrient supply to the fetus. EVT-driven decidual tissue restructuring and vascular adaptation are essential for establishing a functional fetal–maternal interface, to which the decidual microenvironment is thought to contribute substantially. However, the precise impact of the degree of EVT invasion on decidual architecture and immune cell distribution remains poorly understood, underscoring the need to elucidate how varying EVT abundances shape the morphological and cellular landscape of the decidual microenvironment.

**STUDY DESIGN, SIZE, DURATION:**

First-trimester decidual tissue (n = 23, gestational age Weeks 7–9) was analyzed from women undergoing elective terminations of pregnancy between 2011 and 2023. Additionally, hematoxylin and eosin-stained sections from archival specimens (n = 11), obtained from three different sources, were included in the study.

**PARTICIPANTS/MATERIALS, SETTING, METHODS:**

Matched first-trimester *decidua basalis* and *decidua parietalis* samples from the same donors were analyzed through a comprehensive approach combining spatial transcriptomics, histomorphological characterization, and quantitative image analysis. Decidua sections were (i) categorized according to the degree of invasion, (ii) subjected to spatial transcriptomics, including integration with a previously published single-cell RNA-seq dataset, and (iii) quantitatively assessed on the protein level for selected immune cell populations with immunostaining and semi-automated image analysis. The study was complemented by (iv) an observer-based histological evaluation and (v) comprehensive staining series of consecutive decidua sections.

**MAIN RESULTS AND THE ROLE OF CHANCE:**

Analyses revealed a characteristic tissue restructuring of the decidua and distinct spatial patterns of immune cell abundance in relation to the degree of EVT invasion. In strongly invaded decidual areas, we identified regions with pronounced morphological changes—defined as ‘remodeling lesions’. These remodeling lesions typically displayed compromised tissue integrity, eroded blood vessels, extravasal erythrocytes, fibrin deposits, and a distinct gene expression profile, reflecting coagulation, fibrinolysis, and tissue restructuring. While we observed a decline in local immune cell populations—specifically T cells, macrophages, and decidual natural killer cells—with increasing EVT density, neutrophils were almost exclusively located within or in close proximity to remodeling lesions, indicating a substantially reshaped immune landscape.

**LARGE SCALE DATA:**

Spatial transcriptomics data are available in the Gene Expression Omnibus repository under accession number GSE301306.

**LIMITATIONS, REASONS FOR CAUTION:**

Studies using first-trimester placental tissues from elective terminations are inherently limited by surgical disruption of the intact (*in toto*) anatomical architecture of the tissue and the unknown pregnancy outcome. Spatial transcriptomics was performed on a limited number of tissue sections, and histological tissue sections represent just a snapshot, highlighting the limitations of such tissue-based analyses.

**WIDER IMPLICATIONS OF THE FINDINGS:**

While blood leakage into the stromal tissue compartment has typically been documented for pathological conditions—such as large atherosclerotic plaques and tumors—we report such a scenario under physiological conditions for the early invaded decidua. We propose that strong EVT invasion induces remodeling lesions in the *decidua basalis* and also shapes the surrounding immune cell landscape. We further suggest that the occurrence of these remodeling lesions contributes to the establishment of a stable yet flexible basal plate and is thus necessary for a reliable connection between mother and placenta/fetus. It can be speculated that inadequate decidual tissue restructuring and vascular adaptation lead to pregnancy pathologies and complications such as placental abruption.

**STUDY FUNDING/COMPETING INTEREST(S):**

G.M. was supported by the Austrian Science Fund (FWF): PAT9611123. M.G. was supported by the Austrian Science Fund (FWF): 10.55776/P35118 and 10.55776/I6907. This project has received funding from the European Union’s Horizon Europe research and innovation programme under the Marie Skłodowska-Curie grant agreement No 101169308 (funding supported M.G.). M.G., G.M., and J.F. were supported by the Medical University of Graz through the PhD program MolMed. J.F. and G.M. were supported by the COMET center acib: Next Generation Bioproduction (Project #98.311 and #95.802) is funded by BMIMI, BMWET, SFG, Standortagentur Tirol, Government of Lower Austria and Vienna Business Agency in the framework of COMET—Competence Centers for Excellent Technologies. The COMET-Funding Program is managed by the Austrian Research Promotion Agency FFG. The authors declare that they have no conflicts of interest related to this work.

## Introduction

At the contact zone between the placenta and the maternal uterus, fetal cells (extravillous trophoblasts, EVTs) enter and invade the uterine mucosa, referred to as decidua in the pregnant individual, as well as the inner third of the myometrium during early placental development. EVTs invade the stroma and all luminal structures of the decidua, such as glands, spiral arteries, veins, and lymphatic vessels, leading to extensive changes in the decidual tissue architecture to facilitate placental attachment and nutrient supply of the fetus (He *et al.*, 2017; Moser *et al.*, 2017, 2018; Windsperger *et al.*, 2017).

Decidual tissue restructuring and vascular adaptation driven by EVTs are essential processes for a successful pregnancy, to which the decidual microenvironment is thought to contribute critically (Helige *et al.*, 2014; Robertson and Moldenhauer, 2014; Pollheimer *et al.*, 2018; Krstic *et al.*, 2022). The decidual microenvironment is a complex and dynamic network of cells, including stromal, vascular, and immune cells, such as decidual natural killer (dNK) cells, T cells, and macrophages (Vento-Tormo *et al.*, 2018a), that interacts with EVTs and supports the dynamic changes in tissue structure and function. Importantly, although EVTs are semi-allogeneic in normal (own-oocyte) conceptions, these cells are generally tolerated by the local maternal immune system (Benirschke *et al.*, 2012). dNK cells are one of the most prominent and unique immune cell populations of the first-trimester decidua. They support immunotolerance, modulate the invasion of EVTs, and promote the remodeling of uterine spiral arteries (Liu *et al.*, 2021; Wei *et al.*, 2022; Moffett and Shreeve, 2023). Of note, EVTs and tumor cells share many striking characteristics, and both are supported by a similar abetting microenvironment (Krstic *et al.*, 2022).

The process of decidual artery remodeling is described in sequential stages, resulting in dilated arteries with a loss of smooth muscle cells within the wall of the vessels, replacement by fibrinoid, and partial replacement of the endothelium by EVTs (Pijnenborg *et al.*, 2006; Arutyunyan *et al.*, 2023; Greenbaum *et al.*, 2023). As a result, these normally highly contractile arteries transform into rigid tubes, and their lumen widens the closer they get to the intervillous space (Pijnenborg *et al.*, 2006; Burton and Jauniaux, 2023; Greenbaum *et al.*, 2023). Starting with the second trimester of pregnancy, this arterial transformation will eventually serve to provide a high volume of blood flow in combination with low pressure and velocity, required to sustain rapid fetal growth. However, there is still a knowledge gap on how EVTs establish the connection between the uterine circulation and perfusion of the intervillous space of the developing placenta. Arterial remodeling is accompanied by fibrin deposition. Such fibrin deposits are not only present around the arterial walls but can also be frequently observed in various placental compartments at term (Benirschke *et al.*, 2012).

**Figure 1.**
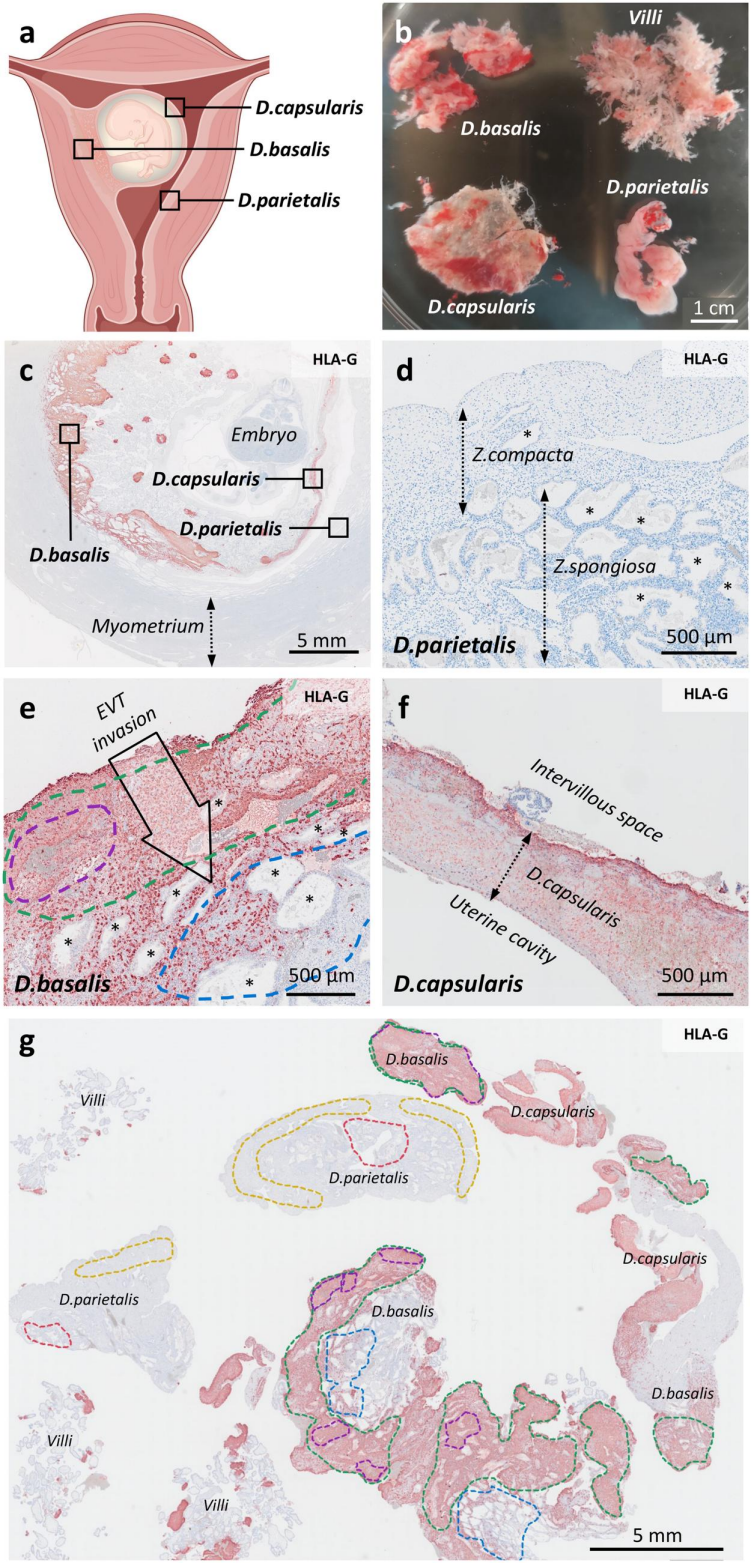
**Types of decidual tissue regions in the first-trimester.** (**a**) Scheme of the developing conceptus within the uterine cavity. The *decidua parietalis* is located distal to the implanting embryo, the *decidua basalis* below it, and the *decidua capsularis* covers it. Created in BioRender. Gauster, M. (2026) https://BioRender.com/pz32fy3. (**b**) Macroscopic appearance of decidual subregions and placental villi. (**c**) Unique archival embryo *in utero* section shows the intact placenta and subregions of the decidua (immunostained for extravillous trophoblast (EVT) marker HLA-G). (**d–g**) Microscopic overview of first-trimester decidua and villi in termination material (immunostained for EVT marker HLA-G): (d) The non-invaded *decidua parietalis* is subdivided into *zona compacta* and *zona spongiosa* and shows dilated, secretory uterine glands (asterisks). (e) EVTs invade into the maternal decidua *basalis* (arrow) and form a gradual invasive front from strong (dashed green line) to weak (dashed blue line) invasion. Remodeling lesions (dashed purple line) are situated in strongly invaded areas and include signs of diminished tissue integrity beside containing less intact nuclei. Uterine glands are marked with asterisks. (f) *Decidua capsularis* usually appears to be highly invaded by EVTs and contains no glands. (g) Areas were annotated based on morphology and degree of EVT invasion. *Decidua parietalis*: (i) *zona compacta* (yellow), (ii) *zona spongiosa* (red); *decidua basalis*: (iii) weak (blue), (iv) strong (green), (v) remodeling lesion (purple). Note that also placental villi are shown, including HLA-G^+^ cell columns. D., decidua; Z., zona. Created in BioRender. Gauster, M. (2026) https://BioRender.com/pz32fy3.

The study of EVT invasion in the first-trimester decidua and the dynamic involvement of various maternal immune cell populations is not only complicated by the availability of decidual tissues but also by the structural and cellular heterogeneity. Notably, the decidual microenvironment is suggested to clearly differ depending on the anatomical region (Vondra *et al.*, 2023). While the site of embryo implantation—which later becomes part of the placental bed, including areas substantially invaded by EVTs—is referred to as the *decidua basalis*, the remaining uterine mucosa not invaded by trophoblasts is called the *decidua parietalis* (Benirschke *et al.*, 2012; Yin *et al.*, 2024). As soon as the embryo has fully embedded into the decidual stroma, the uterine mucosa closes above the implanted embryo and forms a thin layer called *decidua capsularis* that merges with the *decidua parietalis* later in pregnancy (Fig. 1a).

Many studies focused on the overall cell composition of the decidua using single-cell RNA sequencing after dissociation of the tissue (e.g. Suryawanshi *et al.*, 2018; Vento-Tormo *et al.*, 2018a; Nonn *et al.*, 2025) or investigated differences between early and late gestational stages, where the immune cell composition differs considerably (Yang *et al.*, 2019; van der Zwan *et al.*, 2020). While these studies provide valuable insights into the cell populations and their expression patterns, the different spatial regions are rarely considered (or compared), leading to reports with partly varying cell compositions. Very few publications focus on differences in immune cell distribution in relation to the degree of EVT invasion (von Rango *et al.*, 2001).

The research community extensively describes the supporting role of the decidual microenvironment on EVT invasion, while knowledge about the influence of EVT invasion on decidual tissue restructuring and the immune cell landscape is still limited. In this study, we therefore combined state-of-the-art spatial and single-cell transcriptomics analysis with classical histological approaches and quantitative image assessment to comprehensively characterize the human first-trimester decidua in the context of EVT invasion. This synergistic approach enabled a spatially resolved analysis of tissue architecture, gene expression patterns, and immune cell distribution, linking the degree/extent of EVT invasion to morphological and cellular features of the decidual microenvironment. Thereby, we present new details on the establishment of the fetal–maternal interface, especially regarding tissue restructuring and vascular adaptation.

## Materials and methods

### Study design

As this study comprehensively investigated tissue characteristics of the decidua in relation to the degree of EVT invasion, we combined the following approaches (Supplementary Fig. S1): first-trimester decidual tissue was (i) categorized according to the degree of invasion to characterize our donor cohort, (ii) subjected to spatial transcriptomics, including integration with a previously published single-cell RNA-seq dataset (Vento-Tormo *et al.*, 2018a), and (iii) quantitatively assessed on the protein level for selected immune cell populations with immunostaining and semi-automated image analysis. The study was complemented by (iv) an observer-based histological evaluation and (v) a comprehensive staining series of consecutive decidua sections.

### Placental tissue collection

Human first-trimester placental tissue (n = 38 donors, gestational age Week 5–12) of women undergoing voluntary terminations of pregnancy was obtained with ethical approval at Femina Med Center Graz, Austria. Women were included when they met the following inclusion criteria: 18–45 years, no pre-existing conditions, and signed informed consent. Maternal age, BMI, blood group, gestational age, and smoking (self-reported) were documented.

Macroscopic identification of the *villi*, *decidua parietalis*, *basalis*, and *capsularis* was done by an experienced observer. Placental tissue was rinsed in buffered saline, fixed in 4% paraformaldehyde, and embedded in paraffin. Depending on the amount of tissue, more than one formalin-fixed paraffin-embedded (FFPE) block was generated for the same donor (n = 5). A subset of fresh tissue was processed for spatial transcriptomics (see below). Additionally, hematoxylin and eosin (H&E) sections from archival specimens (n = 11, from three different sources, details about donors and processing of tissue unknown, with ethical approval by the Ethics Committee of the Medical University of Graz (Vote No. 28-347 ex 15/16)) from the Department of Cell Biology, Histology and Embryology, Medical University of Graz, were assessed. Paraffin blocks were serially sectioned at 5 µm thickness with a Microm HM355 microtome (Thermo Fisher, Germany); sections were mounted on Superfrost Plus slides (Thermo Fisher, Germany) and further processed for H&E staining. The H&E stains were screened for the presence of donor-matched *decidua parietalis* and *basalis* on the same slide, and thereby tissues of 31 donors were selected and processed for further immune staining (exclusion of 7 donors based on H&E). If more than one block per donor (n = 5) was available, we stained sections from two randomly selected blocks*. Decidua capsularis* was not further analyzed, as it was not consistently present in all cases.

### Ethics

All patients had provided written informed consent. This study was approved by the Ethics Committee of the Medical University of Graz (Vote No. 31-019 ex 18/19) and is in accordance with the Helsinki Declaration of 1964 and its later amendments.

### Definition of areas based on degree of invasion

The areas of interest were annotated using HLA-G-stained sections of decidual tissue (n = 31). *Decidua parietalis* was further subdivided into *zona compacta* and *zona spongiosa*, whereas the *decidua basalis* was subdivided into areas of weak and strong invasion as well as into ‘remodeling lesions’ (always located within strongly invaded areas). The areas were assigned according to morphological criteria (see Table 1 for details on the definition of the areas of interest, including degree of invasion) and were confirmed by an image-based quantitative approach (see below). All areas needed to be present on the same slide to be included. In addition to the seven excluded cases based on H&E staining, further six donors were excluded as not all decidual areas were sufficiently present (exclusion of 6 donors based on HLA-G staining; in total, exclusion of 13 cases, including all cases with gestational age >Week 9). Additionally, two donors were omitted due to a lower gestational age (Weeks 5 and 6), resulting in the final analyzed (n = 23) donor tissues with a gestational age ranging from Weeks 7 to 9 (median of 51 days [Q1–Q3: 49–54]) for further analysis (details of donor cohort in Supplementary Table S1; overview of cohort establishment in Supplementary Fig. S1). Since only two donors with a lower gestational age (Weeks 5 and 6) and none above Week 9 remained, and as the decidua is very dynamic in the early stages of pregnancy, we focused on the narrow gestational window from Weeks 7 to 9 (maternal age range (min–max): 22–39 years). In summary, only matched first-trimester *decidua basalis* and *decidua parietalis* samples from the same donors (n = 23, Supplementary Table S1), but not *decidua capsularis*, were subjected to analysis in this study.

**Table 1.** The defined tissue areas for this study (*decidua parietalis*: (i) *zona compacta*, (ii) *zona spongiosa*; *decidua basalis:* (iii) weak, (iv) strong, (v) remodeling lesion).

*Decidua parietalis*	
*Zona compacta*	No EVTs, dense, compact tissue beyond superficial monolayered uterine epithelium, hardly any glands (if present then with even, round appearance)
*Zona spongiosa*	No EVTs, numerous convoluted irregularly shaped glandular cross sections

*Decidua basalis*	
Weak invasion	Few EVTs (estimated <50% of cells), mostly within *zona spongiosa*, glandular epithelium mostly intact
Strong invasion	Plenty of EVTs (estimated >50% of cells), individual cells clearly recognizable, glandular epithelium often disintegrated
Remodeling lesion	Plenty of EVTs (estimated >80% of cells), individual cells mostly destroyed, tatters of HLA-G–cannot be assigned to single cells, amorph shape of background

EVTs, extravillous trophoblasts.

### Immunohistochemistry and immunofluorescence

All samples (n = 23) were used for immunostaining to validate the expression of the selected markers HLA-G, CD56, CD66b, CD3, CD8, CD14, and CD163. Two selected samples (out of the 23) were used for staining series (CD31, HLA-G, CD235a, fibrin, CD66b, CD3, CD8, CD14, CD163, CD56, and CD11c—additionally to an H&E stain), and five selected samples (out of the 23) were additionally stained for matrix metalloproteinase-1 (MMP1) and CXCL8 as well as processed for immunofluorescence (IF) triple staining (for an overview, see Supplementary Fig. S1).

All antibodies used are listed in Supplementary Table S2 (including: HLA-G—an EVT marker, CD56—a marker of NK cells, CD66b—a marker of neutrophils, CD3—a pan T-cell marker, CD8—a marker of cytotoxic T cells, CD14—a macrophage marker, CD163—an anti-inflammatory macrophage marker, CD31—a marker of vascular endothelial cells, CD235a—a marker of erythrocytes, CD11c—a marker of dendritic cells, fibrin, CXCL8—a pro-inflammatory chemokine, MMP1, and an extracellular matrix (ECM)-degrading enzyme).

FFPE and cryosections (5–7µm) were mounted on Superfrost Plus slides (Thermo Fisher, Germany). FFPE sections were deparaffinized in HistoLab-Clear (Sanova, Austria) and rehydrated in a descending series of alcohol. Antigen retrieval was performed in a microwave in Epitope Retrieval Solution (TRIS (Merck, Germany), EDTA (AppliChem GmbH, Germany) buffer pH 9, with 0.05% Tween 20 (Merck, Germany)) for 15 min at 93°C. Cryosections were fixed in acetone and air-dried. Afterwards, immunohistochemical staining was performed using the UltraVision LP Detection System HRP Polymer Kit (Thermo Fisher Scientific, Germany) as previously described (Guettler *et al.*, 2021). Nuclei were counterstained with Mayer’s hemalum (Thermo Fisher, Germany), and cover slips were mounted with Kaiser’s glycerol gelatin (Merck, Germany). Serial sections were incubated with Negative Control for Rabbit and Mouse Immunoglobulin Fraction (Dako, Agilent, USA) in concentrations corresponding to the primary antibodies. For IF double staining, slides were incubated with blocking solution (Ultravision protein block, Thermo Fisher, Germany) for 7 min at RT before incubation with primary antibody cocktail for 45 min at RT. Afterwards, slides were washed three times in PBS-T before incubation with a secondary antibody cocktail (both ABs 1:200 in PBS) for 30 min at RT. After washing again four times with PBS-T for 10 min, nuclei were stained with DAPI (4',6-diamidino-2-phenylindole, Thermo Fisher, Germany) (1:2000 in PBS), slides were washed three times with aqua dest, and mounted with ProLong Gold Antifade reagent (Invitrogen, Thermo Fisher, Germany).

For IF triple staining, slides were incubated with blocking solution (Ultravision protein block, Thermo Fisher, Germany) for 7 min at RT before incubation with primary antibody cocktail (CD66b and CD3) for 45 min at RT. Afterwards, slides were washed three times in PBS-T before incubation with a secondary antibody cocktail (both ABs 1:200 in PBS) for 30 min at RT. After washing again four times with PBS-T for 10 min, the third primary antibody (CD14, directly conjugated) was applied for 30 min at RT. Slides were washed three times in PBS-T, nuclei were stained with DAPI (Thermo Fisher, Germany) (1:400 in PBS), slides were washed three times with aqua dest, and mounted with ProLong Gold Antifade reagent (Invitrogen, Thermo Fisher, Germany).

All slides were imaged with an Evident Olympus VS200 Slide Scanner (Evident, Germany), and selected slides were imaged with an inverted confocal laser scanning microscope (FV4000, Evident, Japan). Double-stained fluorescence images were acquired using DAPI (nuclei), FITC (auto-fluorescence), Cy3 (CD8), and Cy5 (CD3), as well as Cy3 (CD14) and Cy5 (CD163). Triple-stained fluorescence images were acquired using DAPI (nuclei), FITC (auto-fluorescence), Cy3 (CD3), Cy5 (CD14), and Cy7 (CD66b). Immunohistochemistry (IHC) images were acquired in brightfield using hematoxylin (nuclei) and AEC (3-Amino-9-Ethylcarbazole).

### Image analysis and quantification

Image analysis (n = 23) was performed using Visiopharm VIS (Visiopharm Integrator System) (v2021.09, Visiopharm, Denmark). Annotated areas based on the HLA-G-stained sections (as exemplified in Fig. 1g) were aligned to the H&E-, CD56-, CD66b-, CD163/CD14-, and CD3/CD8-stained sections to assess the same tissue regions in each consecutive image. Nuclei were detected using the nuclei detection app *Nuclei Detection*, *AI (Fluorescence)* for IF images and *Nuclei Detection*, *AI (Brightfield)* for IHC images available within Visiopharm.

Initial total nuclei count on the gathered IHC images stained for HLA-G, CD56, and CD66b, respectively, was performed for all cells by excluding nuclei with a size below 10 µm^2^ and nuclei with a stain below the threshold of <120 in the range of 0–255 in the hematoxylin color deconvolution feature. Not excluded nuclei were dilated by 5.5 µm to perform cell segmentation. A stain intensity feature for the HLA-G, CD56 and CD66b IHC stain, respectively, generated by color deconvolution, was then used to classify these cells as positive, when threshold criteria, >238 in the range of 0–255, were met.

In the double-stained fluorescence images (CD163/CD14; CD3/CD8), detected nuclei with a minimal diameter below 4 µm as well as very weakly stained nuclei, threshold of <7000 in the range 0–65535, were excluded. We identified single-positive cells if the nuclei were attached to or covered by Cy3- or Cy5-positive signals, as well as double-positive cells if nuclei were attached to or covered by Cy3- and Cy5-positive signals. The remaining cells were classified as negative. The detection of positive signals was done using a threshold on each channel, a threshold on a normalized feature generated by background subtraction of the FITC channel (Fuchs *et al.*, 2021), and a threshold on a normalized sharpening feature on the Cy3 and Cy5 channels. The sharpening feature was calculated by subtracting an average of each channel from the original channel. Detected positive signals below a size of 3 µm^2^ were excluded. In order to exclude non-specific positive signals such as erythrocytes, signals that exceeded a threshold in the normalized FITC channel were excluded.

Measurements for the number of total nuclei, single-positive nuclei, and double-positive nuclei were assessed for each area and sample. For comparison and visualization, the number of single-positive or double-positive nuclei was divided by the total number of nuclei. The CD56 IHC staining for three samples did not work, leading to the exclusion of the CD56 IHC staining for these samples. For five donors, sections from two blocks were stained (more than one FFPE block for these donors was available, as described above; for CD56, CD3/CD8, and CD163/CD14), for which the results were averaged.

Additional distance measurements were conducted on triple-stained images (n = 5) to measure distances of CD3^+^ and CD14^+^ cells to the nearest remodeling lesion identified by CD66b^+^ regions. These defined remodeling lesions were manually transferred onto CD56^+^ IHC-stained serial sections of the same tissues (n = 5). Distance measurement of CD56^+^ cells to the nearest remodeling lesion was conducted. This analysis was performed using QuPath version 0.6.0. Remodeling lesion areas were annotated manually and extended by a border up to 600 µm, depending on the tissue area (i.e. the defined measurement area). Vessels were excluded. Cells were detected within these regions using QuPath StarDist version 0.6.0 with a cell expansion of 2 µm; cells with a minimal nucleus diameter below 5 µm were excluded. The detected cells were classified based on intensity measurements. In triple-stained images, cells with a median FITC (autofluorescence) measurement above 5000 were excluded; cells with a median intensity above 4000 and a maximum intensity in the Cy3 channel above 8000 on the nucleus or the cytoplasm were counted as CD3-positive. Cells with a median intensity in the Cy5 channel above 6000 and a maximum intensity above 8000 on the cytoplasm were counted as CD14-positive. In IHC images stained for CD56^+^, detected cells were classified via an object classifier trained on a representative subset.

### Systematic evaluation of histological sections

Image analysis (n = 23) was performed using Visiopharm VIS (v2021.09, Visiopharm, Denmark). Within the defined areas (based on the alignment to serial sections as described above), a histological semiquantitative grading on the H&E stainings (n = 23) was done by trained examiners regarding the content of cellular debris, glandular epithelial integrity, fibrinoid presence, extravasal maternal erythrocytes, and granulocyte presence (by especially evaluating tissue composition, morphology, integrity, and structures). The grading was structured into a score: 0 (no or negligible presence of cells or structures ∼<1% of the examined area), 1 (light presence ∼1–5%), 2 (moderate presence ∼5–10%), and 3 (strong presence ∼>10%). In cases where the examiners could not identify and assess glandular structures clearly, the entry was counted as not applicable (NA), as one cannot distinguish between non-present glands and completely disintegrated glands in an H&E stain. For five donors, sections from two blocks were stained for H&E (more than one FFPE block for these donors was available, as described above), for which the result with the higher score was used. The scores for all 23 donors were visualized in R (v4.4.1, R Core Team (2024), R: A Language and Environment for Statistical Computing. R Foundation for Statistical Computing, Vienna, Austria. <https://www.R-project.org/>) as a stacked bar chart using the R package ggplot2.

Extravasal erythrocytes were counted in H&E-stained sections of the tissue by a histologist (n = 23). Counting was performed in pre-annotated areas of interest (based on the alignment to serial sections as described above). Erythrocytes were identified by color and shape, including cells that were already within degradation processes, e.g. hypertrophy, if an alignment with their erythrocyte origin was possible. Intact vessels were not considered for counting. Erythrocytes in remodeled/impaired vessel structures were included as extravasal erythrocytes. Erythrocyte count was normalized to 1000 µm^2^ for each area of interest. For five donors, sections from two blocks were stained for H&E (more than one FFPE block for these donors was available, as described above); however, one of these sections had to be excluded due to insufficient alignment of the H&E section and the HLA-G section required for quantitative examination. For the remaining four donors, an average was taken of the results.

### Statistical analysis

Analysis results were further analyzed in R. Differences in immune cell populations, HLA-G^+^ cells, and extravasal erythrocyte counts were assessed using Friedman’s non-parametric test, followed by the Durbin-Conover test for *post-hoc* pairwise comparisons with Holm-adjusted *P*-values (R package PMCMRplus v1.9.12). The R package ggstatsplot (v0.12.4) was used for visualization. For the distance measurements, the median distance [µm] for each sample and cell type (CD3^+^, CD14^+^, and CD56^+^) to the remodeling lesions was calculated and visualized using the R package ggstatsplot (v0.12.4) (Patil, 2021). The difference between the distances of the CD3^+^ and CD14^+^ cells within the IF triple-stainings was assessed using a Wilcoxon signed-rank test. To visualize the spatial relationship of CD56^+^ dNK cells, CD3^+^ T cells, and CD14^+^ macrophages to the remodeling lesions, density plots were used (ggplot2 R package, v4.0.1) (Wickham, 2016).

### Single-cell RNA-seq data analysis

The single-cell RNA-seq dataset published by Vento-Tormo *et al.* (2018a,b) was downloaded from ArrayExpress (E-MTAB-6701). We restricted the analysis to samples originating from the decidua, comprising 14 sequencing libraries (seven CD45^+^ and seven CD45^−^) from six individuals for the decidua. A data analysis workflow similar to Gorsek Sparovec *et al.* (2022) was applied. Briefly, Cell Ranger (10x Genomics, v.3.1.0, Netherlands) was used to process each sample. The generated count matrices were further processed in R (v.4.2.1) using the R packages Seurat (Hao *et al.*, 2021) (v.4.2.0) and Harmony (Korsunsky *et al.*, 2019) (v.0.1.0). We kept cells with <20% mitochondrial counts and at least 500 unique features and only included features detected in at least three cells. The workflow included pre-processing (normalization, variable feature identification, scaling, and linear dimensional reduction), Harmony integration, clustering, and visualization via Uniform Manifold Approximation and Projection (UMAP). Resulting clusters were assigned to cell types based on typical markers, with annotations corresponding closely to the decidual cell types defined in Vento-Tormo *et al.* (2018a). We performed filtering steps to exclude clusters/cells deemed as contaminations (Vento-Tormo *et al.*, 2018a) and reintegrated the cleaned data. To facilitate visualization, related cell subpopulations were merged as appropriate, and cell types with <150 cells were excluded. The single-cell data were further used to deconvolve the spatial transcriptomics data, as described below.

### Spatial transcriptomics

*Decidua basalis* and *parietalis* tissues were dissected into cubes with ∼4 mm side length (n = 2 vs 2, tissues from two donors of our donor cohort). Tissue was embedded in OCT (optimal cutting temperature) compound, frozen in isopentane (Sigma-Aldrich, Germany) in liquid nitrogen, and stored at −80°C according to the tissue preparation guide (CG000240 RevA). Frozen samples were cryosectioned at 10 µm thickness at −20°C in a Cryostar NX50 (Thermo Fisher, Germany) according to the tissue preparation guide (CG000240 RevA) and placed on pre-chilled Visium Tissue Optimization Slides (10x Genomics, Netherlands) and Visium Spatial Gene Expression Slide (10x Genomics, Netherlands) within the defined capture areas. *Decidua basalis* and *parietalis* from each placenta were placed into one capture area, respectively (four capture areas in total). The slide was stored at −80°C until further processing.

Tissue optimization for spatial analysis was performed according to the Visium Spatial Gene Expression Reagent Kits—Tissue Optimization User Guide (CG000238, 10x Genomics, Netherlands). For gene expression analysis, sections were fixed and H&E-stained according to the H&E Staining Guide (CG000160 RevB, 10x Genomics, Netherlands) and imaged on an Aperio Scan Scope AT2 System using the Aperio ImageScope software (Leica Biosystems, Germany). Sections were permeabilized for 6 min followed by reverse transcription, second-strand synthesis, denaturation, and cDNA amplification according to the Visium Spatial GEX User Guide (CG000239 Revision D, 10x Genomics, Netherlands). cDNA cycle number determination by quantitative real-time PCR revealed 13–14 cycles for cDNA amplification. Amplified libraries were quantified using the High Sensitivity DNA Bioanalyzer Kit (5067-4626, Agilent, USA). Library fragmentation, end repair, A-tailing, adapter ligation, and indexing were done according to the Visium Spatial GEX User Guide (CG000239 Revision D, 10x Genomics, Netherlands). For sample indexing, 17–18 cycles were performed using the Dual Index Plate TT set A (1000215, 10x Genomics, Netherlands). Size selection and clean-up were performed using SPRIselect reagent (B23318, Beckman Coulter, USA), according to the manufacturer’s instructions. The resulting libraries were verified using the High Sensitivity DNA Bioanalyzer Kit (5067-4626, Agilent, USA) with an average length of 449 bp. The quantity of the libraries was assessed using QuantiFluor ONE dsDNA Dye (E489A, Promega) with the QuantiFluor ONE Lambda DNA (E490B, Promega, USA) on a Quantus^TM^ Fluorometer. Finally, libraries were normalized and pooled according to the manufacturer’s instructions (CG000239 Revision D, 10x Genomics, Netherlands). The pooled library was sequenced on an Illumina Nova Seq SP flow cell in paired-end mode (28/10/10/90).

10x Genomics-based Visium workflow was performed by the Core Facility Molecular Biology at the Medical University of Graz, Austria. Sequencing of the libraries was done by the Next Generation Sequencing Facility at Vienna BioCenter Core Facilities (VBCF), member of the Vienna BioCenter (VBC), Austria. After demultiplexing, FastQ files were used for data analysis.

### Spatial transcriptomics data analysis

The sequencing data were analyzed using Space Ranger (v1.2.2, 10x Genomics, Netherlands). The data were thereby aligned to the human reference genome (GRCh38-2020-A) and quantified. Space Ranger automatically aligned the spots to the corresponding H&E slide images and defined the spots located under the tissue sections. The Space Ranger results were analyzed in R (v4.4.1). Data quality was assessed, and spots with a unique feature count below 600 and mitochondrial count percentage above 30 were filtered. By following the analysis guide from 10x Genomics (https://www.10xgenomics.com/analysis-guides/correcting-batch-effects-in-visium-data, last accessed January 2025), we analyzed the Visium data, including a batch effect correction using the R packages Seurat (Hao *et al*., 2024) (v5.1.0) and Harmony (Korsunsky *et al.*, 2019) (v1.2.1). The FindClusters function was run with the parameter resolution = 0.4. The clustering was annotated based on the corresponding H&E staining, HLA-G expression, and the expression of marker genes. In addition, a manual annotation of the defined areas was carried out using the Loupe Browser (10x Genomics, v7.0.1, Netherlands) (*decidua parietalis*: (i) *zona compacta*, (ii) *zona spongiosa*; *decidua basalis*: (iii) weak invasion, (iv) strong invasion, (v) remodeling lesion; NA—spots not included in the annotation, e.g. due to glandular lumen and folded tissue), informed by the H&E staining and the IHC stainings of the serial sections, and read into R. These annotations were additionally transferred to HLA-G-stained serial sections. Within these annotations, the positive HLA-G-stained area was measured in VIS v2021.09 (Visiopharm, Denmark) and presented in percent of the positive HLA-G area (area-based calculation was used, as this was based on cryosections). The Seurat functions SpatialDimPlot, DimPlot, SpatialFeaturePlot, FeaturePlot, and DotPlot, as well as functions from the ggplot2 R package (v3.5.1) (Wickham, 2016), were used for visualization. For better visualization, we used the parameters pt.size.factor = 1.25, crop = F, and keep.scale = ‘all’ (applied separately for each plotted gene) in SpatialFeaturePlot. To carry out differential expression testing (remodeling lesion vs strong), the Seurat function FindMarkers was applied, and the results were filtered based on the parameters: adjusted *P*-value < 0.05, absolute average log2FC > 0.5, and percentage of cells of the group of interest (pct.1) > 0.3.

The R package spacexr (Cable *et al*., 2022) (v2.2.1) was applied to deconvolve the spatial dataset using the public first-trimester single-cell RNA-seq data (Vento-Tormo *et al.*, 2018a) as a reference (integration according to the protocol provided by 10x Genomics, https://www.10xgenomics.com/analysis-guides/integrating-10x-visium-and-chromium-data-with-r, last accessed January 2025), using the normalized weights to estimate the cell type proportions. To facilitate visualization, related cell subpopulations were merged as appropriate, averaged across areas of interest, and visualized as a stacked bar chart using the ggplot2 R package (v3.5.1).

## Results

### Histological evaluation of the degree of EVT invasion reveals morphologically conspicuous areas

Reliable identification of *decidua basalis*, *parietalis*, and *capsularis* is crucial to comprehensively investigate the characteristics of the decidual microenvironment and architecture in relation to the degree of EVT invasion (Fig. 1a; Supplementary Fig. S1). Due to the surgical procedure, the tissue is normally not preserved *in toto* (i.e. by vacuum aspiration from the uterus, the tissue is disrupted into fragments; Fig. 1b). Only in a unique archival placenta *in utero* specimen, all anatomical regions of the fetal–maternal tissues are presented in their *in vivo* location (Fig. 1c). While the distinction is macroscopically possible for an experienced observer (Yin *et al.*, 2024) (Fig. 1b), we also confirmed the presence/absence of EVTs by immunostaining for the EVT-marker HLA-G (Fig. 1c–g). *Decidua parietalis* was verified by the absence of HLA-G staining in both the *zona compacta*, densely packed with decidual stroma cells, as well as in the *zona spongiosa*, a layer with dilated and irregularly shaped uterine glands (Fig. 1d).

To enable the study of decidual tissue characteristics in relation to EVT abundance, we defined areas of the decidua in our cohort based on the degree of EVT invasion (Fig. 1g; Table 1), as follows: non-invaded *decidua parietalis*: (i) *zona compacta* and (ii) *zona spongiosa* as well as invaded *decidua basalis*: areas of (iii) weak and (iv) strong invasion based on HLA-G staining. Weak and strong degrees of invasion were classified by the extent of EVT abundance (Table 1;  Fig. 1e; Supplementary Fig. S2) and confirmed by quantifying the proportion of HLA-G^+^ cells in all tissue areas (Supplementary Fig. S3a). In all decidua sections (n = 23 with matched *decidua basalis* and *parietalis*), each area was annotated at least once. During this process, we noted (v) areas that were morphologically conspicuous within the areas of strong invasion of the *decidua basalis* (area marked in purple in Fig. 1e and g; Supplementary Fig. S2). Such conspicuous areas were found at least once in all *decidua basalis* samples and included signs of diminished tissue integrity and the presence of cell debris, while HLA-G staining could not be properly attributed to cells. The tissue in this area appeared to contain less intact nuclei, which could not be reliably assigned to cells. Fewer—mostly disintegrated—glandular structures were visible, and the tissue was interspersed with interstitial gaps. Based on the histological appearance, we defined these focal areas as ‘remodeling lesions’. Importantly, all donor tissues (n = 23) contained all five areas of interest present on the same slide.

The weakly invaded areas displayed mostly normal tissue structure (including intact glandular integrity) and almost always coincided with the *zona spongiosa*. Strongly invaded areas presented with diminished tissue and glandular epithelial integrity and increased density of the tissue (Fig. 1e and g; Supplementary Fig. S2). Therefore, it was not possible to clearly distinguish between *zona spongiosa* and *zona compacta* for this area type. Remodeling lesions were situated within strongly invaded areas, mostly in the superficial *decidua basalis* and usually close to anchoring villi (if visible, depending on tissue orientation).

### Spatial transcriptomics maps the tissue structure, the EVT-invaded regions, and immunomodulating mechanisms

**Figure 2.**
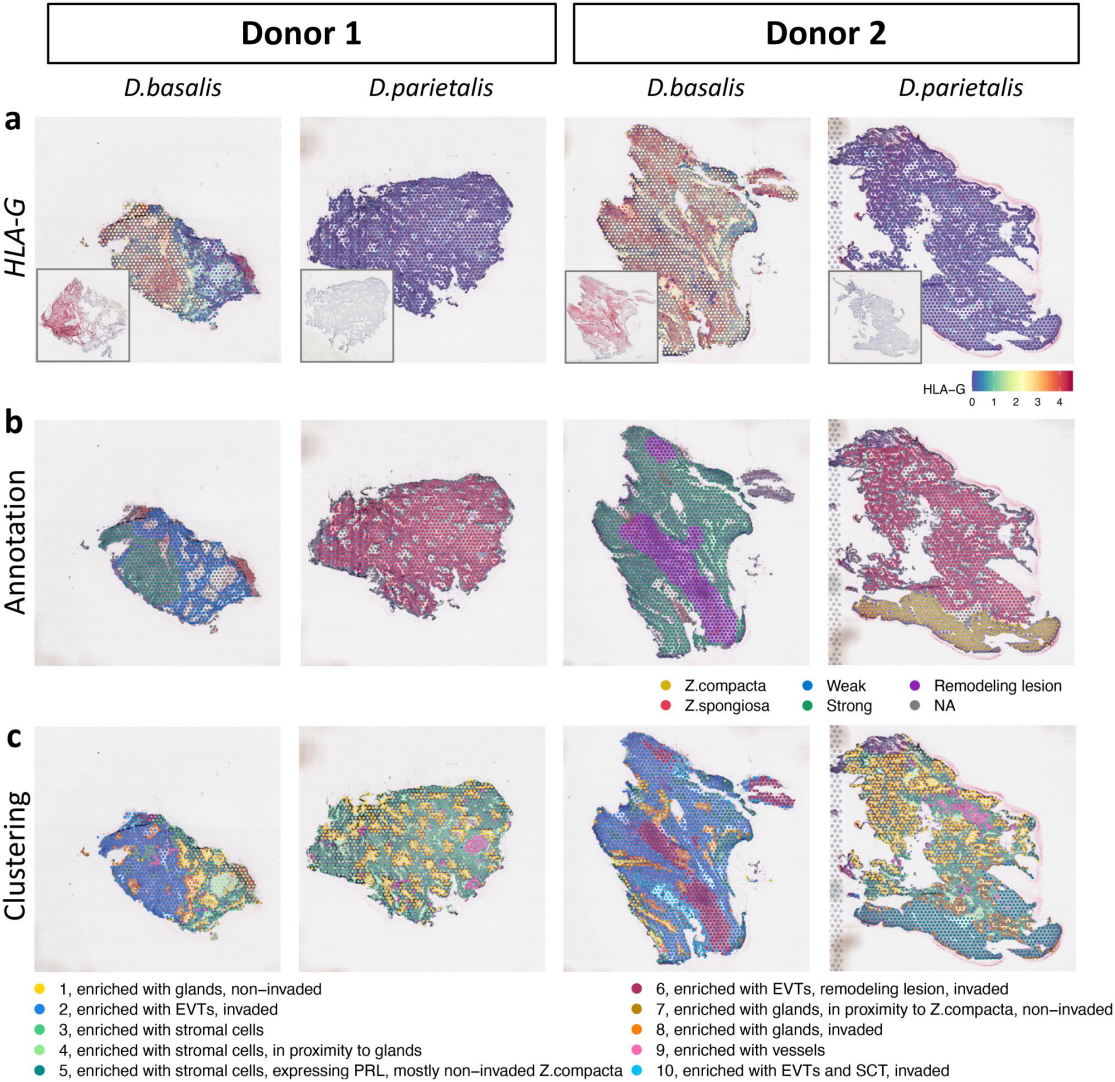
**Spatial transcriptomics visualizes extravillous trophoblast (EVT)-invaded regions and the structure of the tissue.** (**a**) *HLA-G* expression, as a marker for EVTs, maps the EVT-invaded regions (normalized expression; shades from red to blue encode a high to low value range). Small insets show a serial section immunostained for HLA-G (see Supplementary Fig. S4 for all stainings). This shows that the *HLA-G* expression and the HLA-G immunohistochemistry (IHC) staining align very well, revealing two invaded and two non-invaded tissue sections. (**b**) Areas annotated by histologists—*decidua parietalis*: (i) *zona compacta* (yellow), (ii) *zona spongiosa* (red); *decidua basalis*: (iii) weak invasion (blue), (iv) strong invasion (green), (v) remodeling lesion (purple); NA—spots not included in the annotation (gray), e.g. due to glandular lumen and folded tissue. Area annotations were based on the HLA-G staining and the architectural context provided by the hematoxylin and eosin (H&E) stainings. (**c**) Spatial distribution of the clustering results (for cluster definition/annotation, see Supplementary Fig. S5), highlighting the heterogeneous tissue architecture by revealing EVT-invaded regions, decidual stroma, glandular structures, and vascular-rich regions, among others, that align well with the area annotations in (b). *Decidua basalis* and *parietalis* from two donors. D., decidua; Z., zona; SCT, syncytiotrophoblast; *PRL*, gene encoding prolactin.

To gain deeper insight into decidual architecture, spatial transcriptomics using the Visium platform from 10x Genomics was performed on four decidua sections (matched *decidua basalis* and *parietalis* from two different donors, annotated with the defined areas of the decidua). For additional tissue characterization, H&E staining of the same sections and IHC (HLA-G, KRT7, and CD34) of serial sections were carried out (Supplementary Fig. S4a). Spatial transcriptomics distinctly mapped the invading EVTs and the structure of the tissue, such as glands, vessels, and anatomical regions (Fig. 2a–c). The expression level of *HLA-G* coincided with the HLA-G immunostaining (Fig. 2a;  Supplementary Fig. S4a), uncovering an invasive front for Donor 1 and a completely invaded sample for Donor 2.

We used the H&E and IHC stainings to manually annotate areas (as described above) within the four tissue sections (Fig. 2b). The strongly invaded Donor 2 sample showed the presence of a remodeling lesion (Fig. 2b and c; Supplementary Fig. S4b). The highest expression levels of *HLA-G* were observed in the strongly invaded and remodeling lesion areas (Supplementary Fig. S3b), which aligned with the proportion of the HLA-G^+^ area in the HLA-G IHC staining of serial sections (*zona compacta* and *spongiosa*: each 0%; weak invasion: 9%; strong invasion 46%; remodeling lesion: 46%; based on the HLA-G staining shown in Supplementary Fig. S4a). Importantly, we identified 10 clusters based on gene expression patterns (Supplementary Fig. S5a), annotated these clusters based on marker expression (Supplementary Fig. S5b), and visualized their spatial distribution (Fig. 2c). This agreed with the immunostainings and the manually annotated areas and also revealed further insights: a cluster (#5), enriched with *PRL*-expressing decidual stromal cells (DSCs), was mostly present in the non-invaded *zona compacta* (Supplementary Figs S5a and b and S6a and c). While the expression of the chemokine *CXCL14* in epithelial cells has been acknowledged before (Mokhtar *et al.*, 2010), further investigation into spatial expression patterns of the clusters demonstrated that this marker particularly highlights the decidual glands on a transcriptomic level (Supplementary Fig. S6b). A distinct cluster (#10) displayed expression of EVT and syncytiotrophoblast (SCT) markers and was localized to areas that also included SCT fragments in the H&E stain. This is indicated by the spatial expression pattern of SCT marker genes, which clearly highlighted the SCT fragment-containing areas (Supplementary Fig. S7a–d). Such SCT fragments were also observed in the superficial *decidua basalis*, which is a frequently reported feature (Nonn *et al.*, 2025). The remodeling lesion area presented with a distinct expression pattern, forming a separate cluster (#6).

Immunomodulating processes are essential for establishing immunotolerance toward EVTs during early pregnancy (Vento-Tormo *et al.*, 2018a). *HLA-G*, several inhibitory ligands, and other immunomodulatory molecules known to be expressed by EVTs, such as *ACKR2—*a decoy receptor that scavenges and internalizes inflammatory chemokines—support these processes (Teoh *et al.*, 2014; Vento-Tormo *et al.*, 2018a; Gowhari Shabgah *et al.*, 2022; Krstic *et al.*, 2022). Here, we showed that EVTs express (and therefore areas invaded by EVTs display expression of) *HLA-G*, *ACKR2*, *CD276 (B7-H3)*, *CD274 (PD-L1)*, *PDCD1LG2 (PD-L2)*, *PVR (CD155)*, and *PTGES* (Prostaglandin E Synthase involved in PGE_2_ synthesis) (Supplementary Fig. S8a and b). Comparing the remodeling lesions to the strongly invaded areas revealed a distinct expression profile, including upregulated genes such as *CXCL8*, *MMP1*, *MMP10*, and *CYGB*, which are associated with immune response, tissue remodeling, and oxidative stress (Supplementary Fig. S8c). This expression profile was also observed in the cluster-based annotation, showing that cluster #6 (remodeling lesion) and the manually annotated remodeling lesion had a similar expression pattern (Supplementary Fig. S8d). Most importantly, the spatial transcriptomics data confirmed that our histological annotations were indeed linked to distinct gene expression profiles, and that the clustering results reflected the structure of the tissue.

### Immune cell abundance profoundly differs in relation to the degree of invasion and defined areas of the decidua

Given the immunomodulatory properties of EVTs, we next sought to investigate their role in shaping the local immune environment by analyzing how immune cell populations are distributed in the decidua in relation to EVT invasion. We used serial sections aligned to our HLA-G stainings from our decidua cohort (Fig. 1g), enabling us to transfer the defined areas.

**Figure 3.**
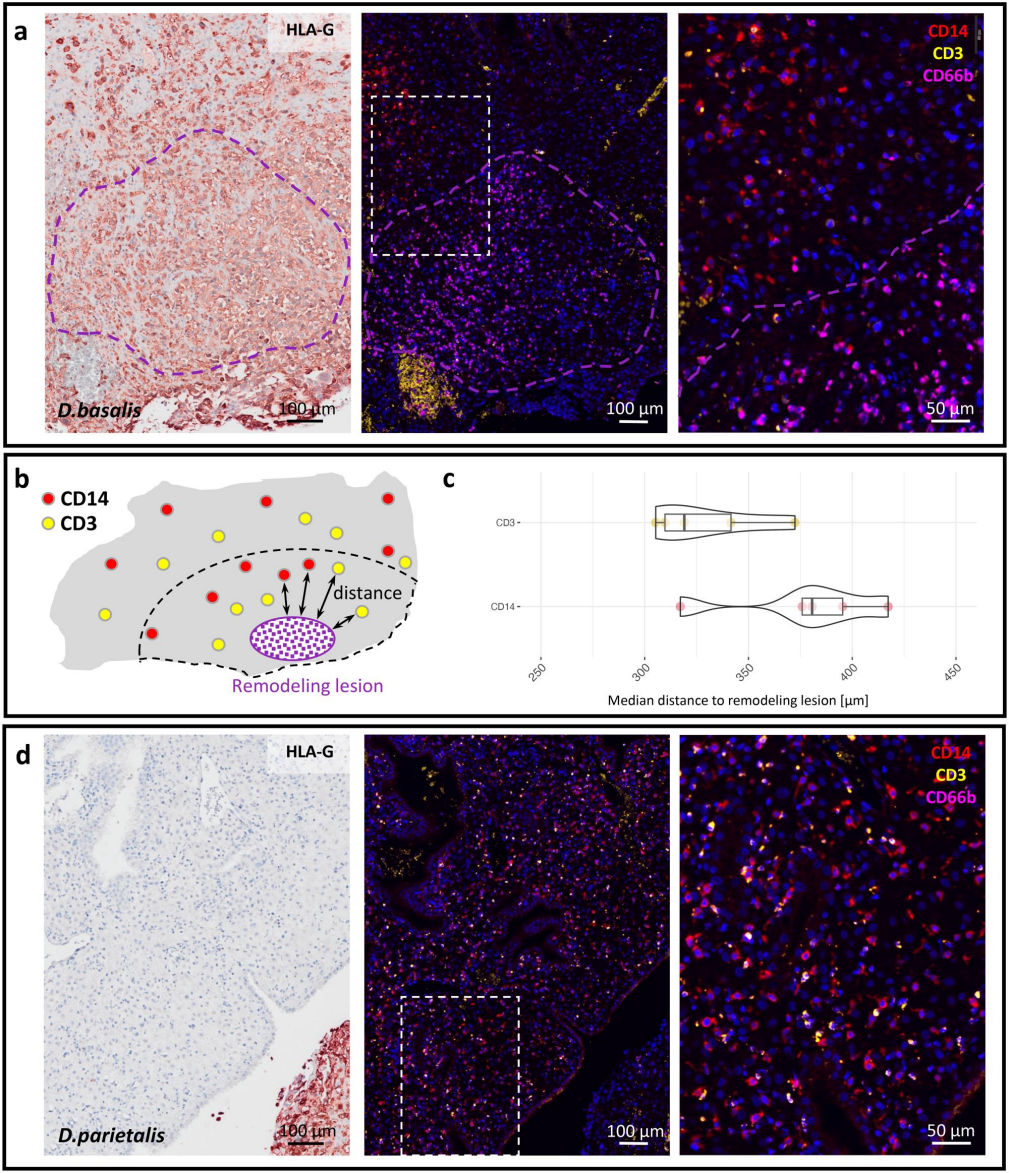
**Detection of three main immune cell types in the decidua and their spatial relationships, with a focus on their proximity to remodeling lesions.** (**a**) Serial sections were stained by immunohistochemistry (IHC, first column) or immunofluorescence (IF) triple-staining (second and third columns) to visualize extravillous trophoblast (EVT) invasion (HLA-G) and immune cell distribution of neutrophils (CD66b^+^, magenta), T cells (CD3^+^, yellow; please note that erythrocytes produce nonspecific signal in this channel due to autofluorescence), macrophages (CD14^+^, red). Within a remodeling lesion (in the strongly invaded *decidua basalis*), highlighted by the dashed purple line, extravasal neutrophils are present, whereas T cells and macrophages locate distantly. (**b**) Schematic showing the quantitative distance measurements of CD3^+^ and CD14^+^ cells to the nearest remodeling lesion within a defined measurement area (dashed black line). (**c**) Boxplots visualizing the median distance [µm] of CD3^+^ T cells and CD14^+^ macrophages to remodeling lesions (n = 5). (**d**) Within *decidua parietalis*, T cells and macrophages are evenly distributed throughout the decidual stroma, and neutrophils are not visible. Nuclear counterstain with hematoxylin for IHC or DAPI for IF. White insets indicate the enlargements shown in the third column.

Triple IF stainings—neutrophils (CD66b^+^), T cells (CD3^+^), and macrophages (CD14^+^)—were performed on five selected donor tissues of our donor cohort. In the *decidua basalis*, extravasal neutrophils were clearly present within all remodeling lesions, whereas T cells and macrophages were located distantly from the lesions (Fig. 3a). To investigate their spatial relationships in relation to the remodeling lesions, we calculated the distances from the remodeling lesions to the CD3^+^ T cells and CD14^+^ macrophages, respectively (Fig. 3b). The results, while not significant, indicated a trend that T cells were located closer to the remodeling lesions (and hence to the invasive front) than macrophages (*P*-value = 0.0625; Fig. 3c). The distances of CD66b^+^ neutrophils to remodeling lesions were not measured, as these cells are predominantly located within the remodeling lesions. Within the *decidua parietalis*, T cells and macrophages were evenly distributed throughout the decidual stroma, and neutrophils were only present within decidual blood vessels (Fig. 3d). Additionally, we quantified the distances of CD56^+^ dNK cells to the remodeling lesions based on IHC stainings (n = 5), showing that these cells also preferably located distantly (Supplementary Fig. S9a–c). Distance distribution profiles of the three cell types (CD3^+^ T cells, CD14^+^ macrophages, and CD56^+^ dNK cells) showed that cells were more concentrated at higher distances away from the remodeling lesions (Supplementary Fig. S9d). However, we noted that while the presence of CD3^+^ T cells and CD14^+^ macrophages in remodeling lesions was scarce, dNK cells and neutrophils were commonly observed within these lesions (Fig. 3a; Supplementary Fig. S9a); notably, in contrast to dNK cells, neutrophils were specifically detected within the remodeling lesions.

**Figure 4.**
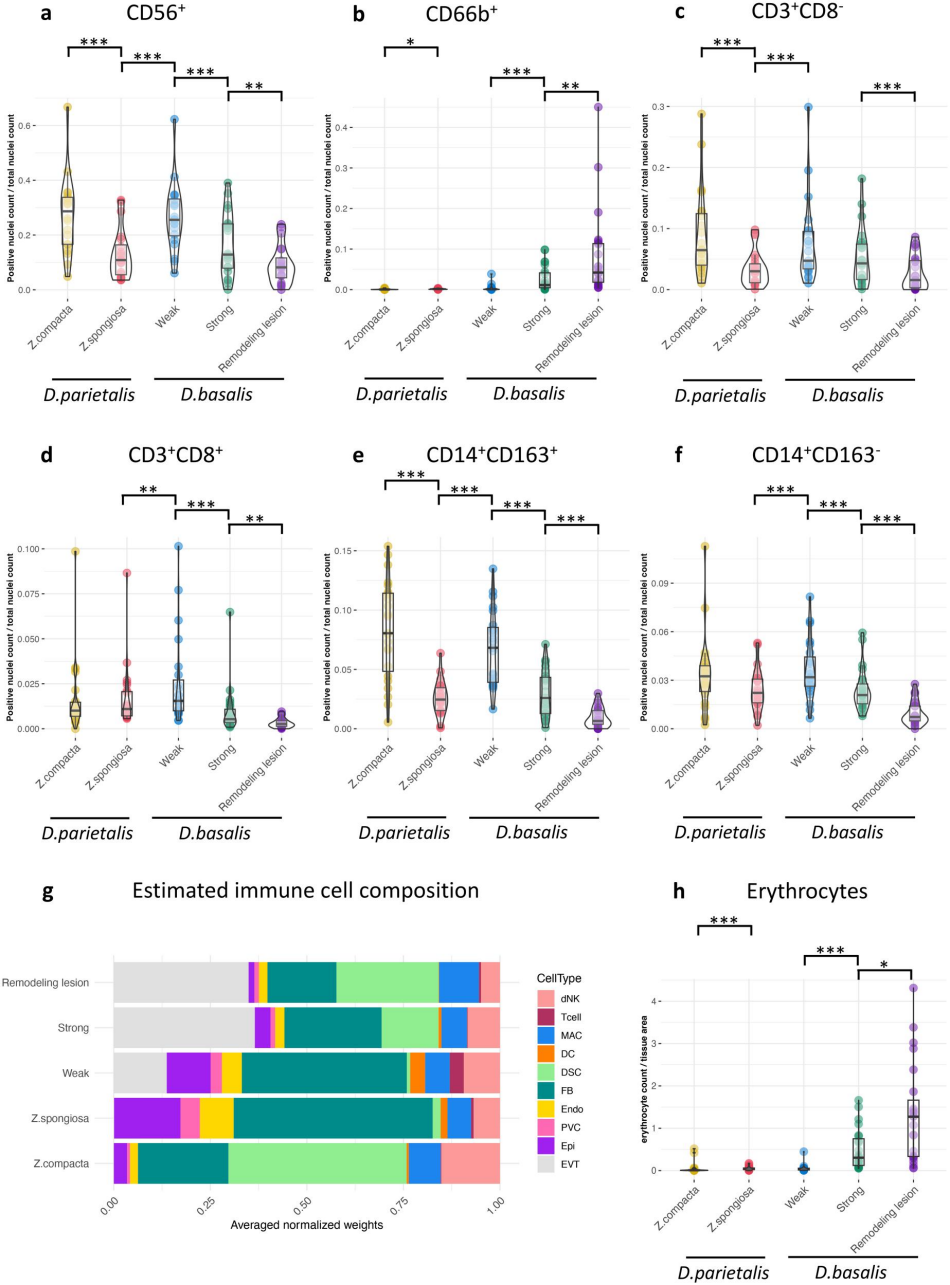
**(Immune) cell populations in the human first-trimester decidua.** Immune cell abundance across the defined tissue areas (*decidua parietalis*: (i) *zona compacta*, (ii) *zona spongiosa*; *decidua basalis*: (iii) weak invasion, (iv) strong invasion, (v) remodeling lesion). The number of single-positive or double-positive nuclei divided by the number of total nuclei for (**a**) CD56^+^ decidual natural killer (dNK) cells, (**b**) CD66b^+^ neutrophils, (**c**) CD3^+^CD8^−^, and (**d**) CD3^+^CD8^+^ T cells as well as (**e**) CD14^+^CD163^+^ and (**f**) CD14^+^CD163^−^ macrophages, quantitatively assessed in immunohistochemistry/immunofluorescence (IHC/IF) images (n = 23). (**g**) Estimated immune cell composition (averaged normalized weights) based on the spatial transcriptomics data (*decidua basalis* and *parietalis* from two donors) integrated with a publicly available single-cell RNA-seq dataset (Vento-Tormo *et al.* (2018a,b), averaged across the defined tissue areas and visualized as stacked bar chart. (**h**) Erythrocyte count per 1000 µm^2^ tissue area on hematoxylin and eosin (H&E)-stained sections (n = 23). *Adjusted *P*-value ≤ 0.05; **adjusted *P*-value ≤ 0.01; ***adjusted *P*-value ≤ 0.001. To improve readability, only significant differences between adjacent groups are shown (results of all comparisons are listed in Supplementary Table S2). dNK, decidual natural killer cells; Tcell, T cells; MAC, macrophages; DC, dendritic cells; DSC, decidual stromal cells, FB, decidual fibroblasts; Endo, endothelial cells; PVC, perivascular cells; Epi, epithelial cells; EVTs, extravillous trophoblasts; D., decidua; Z., zona.

To quantitatively assess the distribution of the immune cell populations in the decidua (and to further confirm our observations above), we subsequently performed single IHC or double IF stainings for the key immune populations in the decidua to evaluate their spatial localization and abundance in relation to the degree of EVT invasion (n = 23). Thereby, we quantitatively assessed nuclei counts of CD56^+^ dNK cells, CD66b^+^ neutrophils, CD3^+^CD8^−^ and CD3^+^CD8^+^ T cells, CD14^+^CD163^−^ and CD14^+^CD163^+^ macrophages (Supplementary Figs S10 and S11). This revealed profound differences in the immune cell abundance in relation to the degree of EVT invasion as well as between the non-invaded *decidua parietalis* areas, namely, *zona compacta* and *zona spongiosa* (Fig. 4a–f; results/*P*-values of all comparisons are listed in Supplementary Table S3). In line with current literature, our results showed that dNK cells are the most abundant immune cell type across all areas (for the typically assessed immune cell types, specifically T cells, macrophages, and dNK cells) (Fig. 4a) (Ander *et al.*, 2019; Yang *et al.*, 2019; Xu *et al.*, 2021).

Interestingly, most assayed immune cell populations (CD56^+^ dNK cells, CD3^+^CD8^−^ and CD3^+^CD8^+^ T cells, CD14^+^CD163^−^ and CD14^+^CD163^+^ macrophages) decreased with the degree of invasion (weak invasion > strong invasion > remodeling lesions), alongside the observed morphological changes (Fig. 4a and c–f). However, while dNK cells were reduced in numbers in remodeling lesions, they still persisted in substantial numbers within these lesions as described above (median proportion 8.2%; Fig. 4a). However, the non-invaded *zona spongiosa* of the *decidua parietalis* showed a significantly lower number of these assayed immune cells compared to the weakly invaded areas, indicating that immune cells accumulate along the invasive front. In contrast, the number of CD66b^+^ neutrophils significantly increased in heavily invaded areas, i.e. remodeling lesions and strongly invaded areas (weak invasion < strong invasion < remodeling lesions) (Fig. 4b).

The *zona compacta*, as the uppermost layer of the *decidua parietalis*, needs to provide sufficient defense against infections but is also the first contact point for the invading EVTs. Within the *decidua parietalis*, the *zona compacta* showed a significantly higher number of CD56^+^ dNK cells, CD3^+^CD8^−^ T cells, and CD14^+^CD163^+^ macrophages in comparison to the *zona spongiosa* (Fig. 4a and c–f). With the exception of the cytotoxic CD3^+^CD8^+^ T cells and CD66b^+^ neutrophils, the *zona compacta* showed the highest median immune cell proportions of all decidual areas.

Integration of our spatial transcriptomics data with a publicly available single-cell RNA-seq dataset (Vento-Tormo *et al.*, 2018a) confirmed overall trends of the quantitatively assessed IHC/IF images (Fig. 4g, spatial distribution patterns of estimated proportions for EVTs, epithelial cells, and dNKs in Supplementary Fig. S12a–c). In particular, the *zona compacta* showed a higher proportion of immune cells compared to the *zona spongiosa*, while the strongly invaded and remodeling lesion areas exhibited a decrease in immune cells compared to the weakly invaded areas. Of note, the immune cell proportions may likely be underestimated due to methodological differences and sensitivity issues. Such methodological differences might explain the satisfactory—but not perfect—alignment between the spatial distribution of dNK cells and the corresponding immunostaining. In less-dense tissue areas, transcriptomics analysis appears to underestimate dNK cell presence (Supplementary Fig. S12c and d). Furthermore, compared to the *zona compacta*, the proportion of epithelial cells was found to be higher in the *zona spongiosa*, while the number of the more differentiated PRL-expressing decidual stromal cells (DSC) was estimated to be lower (Fig. 4g;  Supplementary Figs S6a and c and S12b). These observations are in line with recent research (Vento-Tormo *et al.*, 2018a) and coincide with the fact that the *zona spongiosa* is characterized by a large number of uterine glands.

### Extravasal erythrocytes in the tissue are associated with a high degree of invasion

**Figure 5.**
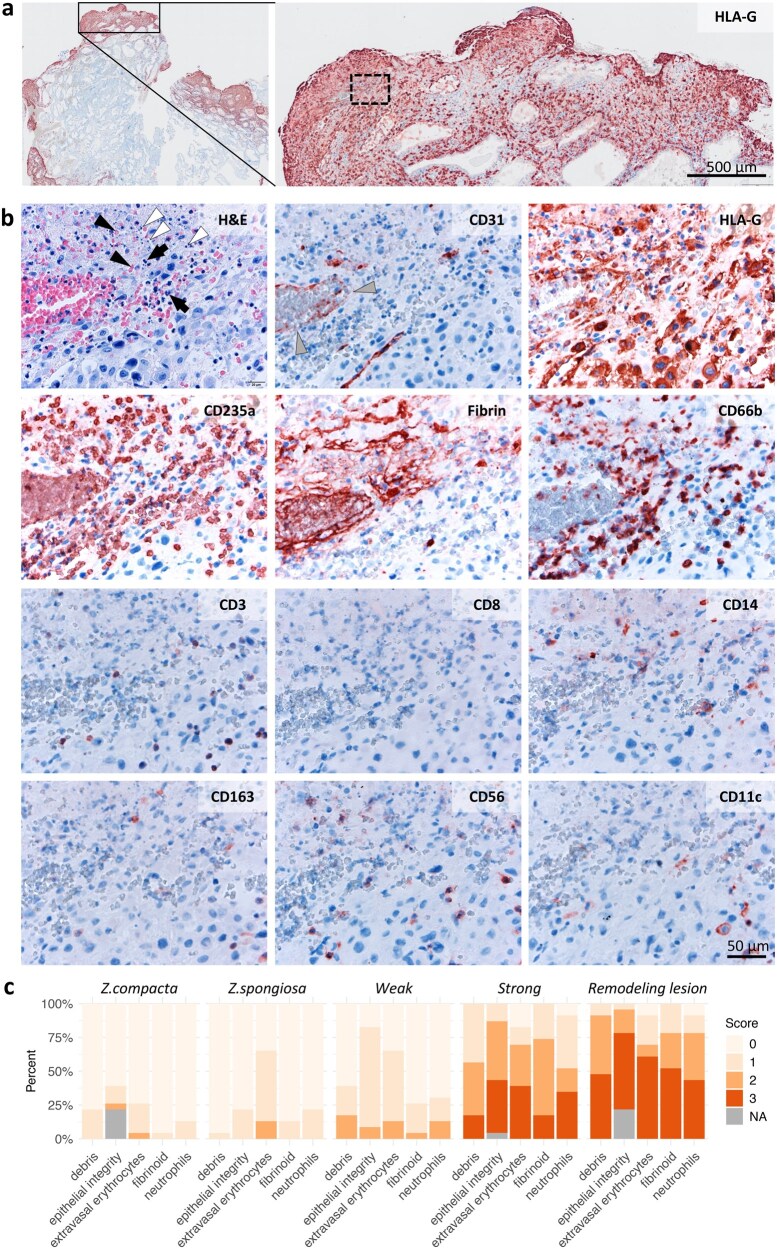
**Staining series of a representative remodeling lesion.** (**a**) Overview of the assessed *decidua basalis* immunostained for HLA-G. Black insets highlight the assessed strongly invaded area with a remodeling lesion (part of the remodeling lesion is further enlarged with a dashed black inset). (**b**) The remodeling lesion is further characterized in serial sections stained by hematoxylin and eosin (H&E) and immunostained for CD31 (endothelium), HLA-G (extravillous trophoblasts (EVTs)), CD235a (erythrocytes), fibrin, CD66b (neutrophils), CD3 (T cells), CD8 (cytotoxic T cells), CD14 (macrophages), CD163 (macrophage differentiation marker), CD56 (natural killer (NK) cells), CD11c (dendritic cells) (exemplified shown for one donor of n = 2). Nuclear counterstain with hematoxylin. The images reveal debris (white arrowheads), fibrin-type fibrinoid deposits, interstitial gaps, interrupted endothelial lining (gray arrowheads), extravasal erythrocytes (black arrowheads), presence of neutrophils (black arrows in H&E image, upper left), and low numbers of immune cells. These parameters were (**c**) systematically assessed in our cohort (n = 23) based on the H&E stainings across the defined tissue areas (*decidua parietalis*: (i) *zona compacta*, (ii) *zona spongiosa*; *decidua basalis*: (iii) weak invasion, (iv) strong invasion, (v) remodeling lesion). In particular, the remodeling lesion areas exhibited high percentages for all parameters. A histological semiquantitative score (0—no or negligible presence of cells or structures ∼<1% of the examined area, 1—light presence ∼1–5%, 2—moderate presence ∼5–10%, and 3—strong presence ∼>10%, NA—examiners could not identify and assess glandular structures clearly) assigned by trained examiners was visualized as stacked bar chart. Z., zona.

Intrigued by the decrease of most immune cell types in the strongly invaded and remodeling lesion areas (summarized as heavily invaded areas), we carried out staining series on consecutive serial decidua tissue sections for H&E and 11 markers (CD66b—neutrophils, fibrin, CD235a—erythrocytes, CD31—endothelial cells, HLA-G—EVTs, CD3—T cells, CD8—T-cell differentiation marker, CD14—macrophages, CD163—macrophage differentiation marker, CD56—NK cells, and CD11c—dendritic cells; Fig. 5a and b; Supplementary Fig. S13; for negative controls, see Supplementary Fig. S14). The staining series revealed debris and fibrin deposits in strongly invaded and remodeling lesion areas, as well as eroded endothelial linings of decidual vessels, interstitial gaps, extravasal erythrocytes, and the presence of neutrophils. Extravasation is supported by a discontinuous endothelial lining of the decidual blood vessel (Fig. 5b, CD31 staining). The occurrence of these characteristics was mostly restricted to the remodeling lesions. However, since remodeling lesions merge into strongly invaded areas without strict boundaries, some characteristics were also observed to a lesser extent in the strongly invaded areas. Furthermore, the staining series visually demonstrated the low/reduced number of macrophages, dNK, and T cells (Figs 4 and 5b) in these heavily invaded areas.

This prompted us to systematically evaluate our cohort based on the H&E stainings for a number of remodeling lesion-specific parameters (debris, fibrinoid, epithelial integrity, extravasal erythrocytes, and neutrophils) (Fig. 5c). The results of this semi-quantitative observer-based evaluation further confirmed the observations of the staining series, with, in particular, the remodeling lesion areas exhibiting high percentages for the maximum rating across all parameters. Of note, we also found such remodeling lesion areas in other archived first-trimester decidua samples obtained in previous decades (Supplementary Fig. S15a–c). The presence of the extravasal erythrocytes was additionally quantitatively confirmed by counting these cells on H&E stainings (Fig. 4h), which clearly revealed a significant increase in heavily invaded areas (weak invasion < strong invasion < remodeling lesions) (results/*P*-values of all comparisons are listed in Supplementary Table S3).

We therefore propose that the integrity of maternal blood vessels becomes compromised during EVT invasion, with erythrocytes and potentially circulating maternal immune cells, especially neutrophils, leaking into the adjacent stroma. Furthermore, the remodeling lesions (and partly the strongly invaded areas) exhibited a distinct expression pattern of genes involved in the coagulation cascade and fibrinolysis (e.g. *PLAT*), oxidative stress (e.g. *HMOX1*), ECM remodeling and tissue repair (e.g. *MMP1* and *MMP10*), angiogenesis and vascular regulation (e.g. *VEGFA*), immune response and neutrophil-related processes (e.g. *CXCL8* and *CSF3R*) (Supplementary Fig. S16a). While several of these genes (e.g. certain MMPs such as *MMP2*) were expressed by EVTs, we suspect that the expression of a number of these genes originated from extravasal neutrophils (e.g. *S100A8* and *S100A9*) and/or other cell types (e.g. the neutrophil chemoattractant genes *CXCL1* and *CXCL8* as well as the stress-related heme oxygenase gene *HMOX1*) responding to the formation of the remodeling lesion (Supplementary Fig. S16a and b). Importantly, we detected the expression of neutrophil markers/neutrophil-related processes in the remodeling lesion area also using spatial transcriptomics (Supplementary Fig. S16a). An exemplary staining series of H&E and HLA-G, CXCL8 and MMP1 within a remodeling lesion and *decidua parietalis*, respectively, is presented in Supplementary Fig. S17a. CXCL8 clearly highlights the remodeling lesions. Additionally, the spatial gene expression patterns of selected remodeling lesion markers (*CXCL8*, *MMP1*, *PLAT*, *HMOX1*, *MMP10*, *VEGFA*, and *CSF3R*) localized in the spatial transcriptomics data are shown in Supplementary Figs S17b and S18.

## Discussion

**Figure 6.**
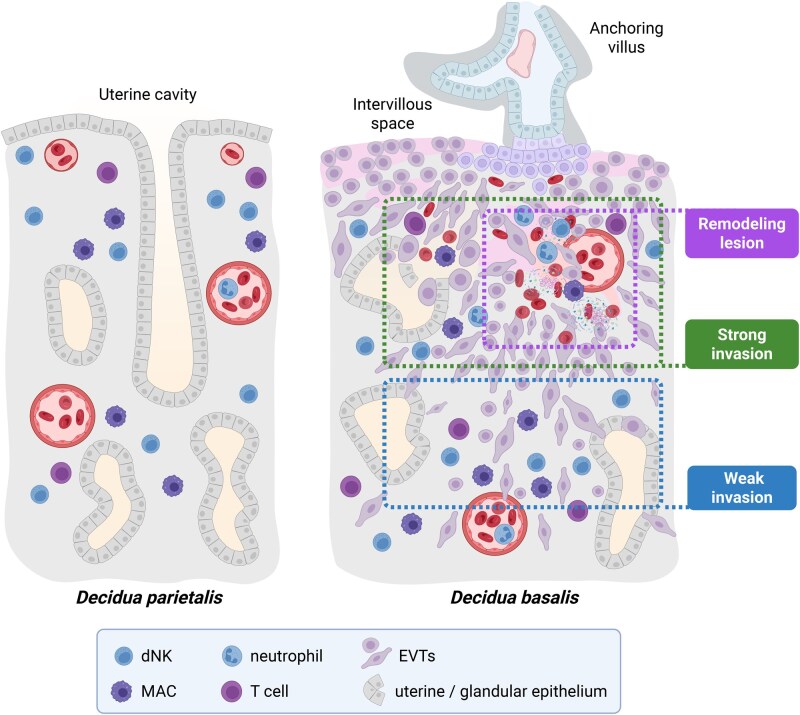
**Remodeling lesions are a common feature of the *decidua basalis*, while they are absent in the *decidua parietalis*.** These lesions are characterized by maternal blood leaking into the decidual stroma (including maternal immune cells such as neutrophils), debris, and fibrin deposits. dNK, decidual natural killer cells; MAC, macrophages; EVTs, extravillous trophoblasts. Created in BioRender. Gauster, M. (2026) https://BioRender.com/0ixjnyu.

In this study, we observe regions in the invaded *decidua basalis* with pronounced morphological changes that we define as ‘remodeling lesions’. Although the process of decidual vascular remodeling during placentation has been described as highly coordinated (Greenbaum *et al.*, 2023), we show that this process is also accompanied by leakage of maternal blood into the surrounding tissue, causing a cascade of events that include the emergence of cell debris, formation of fibrin deposits, and initiation of dynamic tissue remodeling processes (Fig. 6).

We recently described extravasal maternal erythrocytes in interstitial gaps of invaded *decidua basalis* samples (Lyssy *et al.*, 2025). This prompted us to evaluate decidual tissue characteristics in more detail, also in relation to an immunological context. Our results here show that remodeling lesions, alongside maternal blood leakage, appear to be a common feature of the (early) *decidua basalis*, while they are absent in the *decidua parietalis*, which points to a clear association with EVT invasion. In addition to the presence of extravasal maternal erythrocytes, the occurrence of cell debris, fibrin-type fibrinoid deposits, eroded endothelial linings, and pronounced neutrophil niches are characteristics of the heavily invaded areas, especially of the remodeling lesions. We further observe profound differences in the immune cell abundance in relation to the degree of EVT invasion, indicating a substantially reshaped immune cell landscape.

While blood leakage into the tissue has typically been documented for pathological conditions, such as large atherosclerotic plaques and tumors (Humar *et al.*, 2022), we report such a scenario under physiological conditions for the early fetal–maternal interface. As it is known that EVT-derived factors lower endothelial barrier function (Wang *et al.*, 2004) and that EVTs eventually replace the endothelial lining (Greenbaum *et al.*, 2023), we believe that endothelial disruption in these areas enables extravasation of blood cells. Activation of the coagulation system and fibrin deposits are a near-universal aspect of any vessel disruption (Luyendyk *et al.*, 2019). In this study, we show extensive fibrin deposits around eroded maternal decidual vessels, in addition to the characteristic gene expression pattern of the remodeling lesion area in the spatial transcriptomics data, indicative e.g. for coagulation cascade, fibrinolysis, and tissue remodeling. Therefore, we suggest that this vessel erosion in the heavily invaded areas is crucial for tissue remodeling processes and for providing placental structural and vascular support throughout pregnancy. As fibrin is known to build up in the *decidua basalis* during normal pregnancy (Kaufmann *et al.*, 1996; Burton *et al.*, 2025), we further propose that the compromised integrity of the vessels crucially contributes to the generation of fibrin deposits. The literature extensively reports that invaded vessels are surrounded by acellular fibrinoid material (Kam *et al.*, 1999; Craven *et al.*, 2002; Pijnenborg *et al.*, 2006; Burton *et al.*, 2025). Craven *et al.* (2002) describe intravenous blood clots within dilated and invaded decidual veins and fibrin deposits in venous walls. Several studies report the physiological changes of spiral arteries in the *decidua basalis* (trophoblast-associated remodeling), especially the replacement of the arterial media with fibrinoid material (Meekins *et al.*, 1997; Kam *et al.*, 1999; Pijnenborg *et al.*, 2006). In general, fibrinoid material is classified into fibrin-type or matrix-type fibrinoid (Kaufmann *et al.*, 1996). While fibrin-type fibrinoid is a product of the coagulation cascade, matrix-type fibrinoid is produced by EVTs. Our antibody in the staining series specifically detects fibrin-type fibrinoid, which further supports our findings of eroded uterine blood vessels. Given the proximity of EVTs, it is likely that the fibrinoid material in the H&E staining comprises a mixture of both types, as previously suggested (Craven *et al.*, 2002). Such a mixture may be required to provide both flexibility and stability and eventually the required support for a remodeled vascular system. We recently proposed that leukocyte-associated immunoglobulin-like receptor 2 expression of EVTs may be instrumental in these processes, as it could bind and sequester collagen, which in turn may modulate the activation of extravasated platelets (Lyssy *et al.*, 2025).

Support for such remodeling lesions comes from a publication describing the occurrence of CD45^−^ apoptotic cells only in the *decidua basalis* (von Rango *et al.*, 2003). Indeed, the authors report increased apoptosis in strongly invaded areas, along with a decrease in CD56^+^ NK cells, and propose that local maternal immune cells and their cytokine release regulate EVT invasion and apoptosis. Furthermore, apoptotic signaling pathways may be relevant in decidual tissue remodeling by influencing ECM turnover and the clearance of cellular debris and could be assessed in future studies.

With respect to the decidual cell composition, especially frequencies of immune cells, reported numbers vary considerably. Numerous factors cause this variation, including technological differences (Krop *et al.*, 2022) and gestational age (van der Zwan *et al.*, 2020; Greenbaum *et al.*, 2023). As we believe that gestational age has a large influence on the cell composition, we want to emphasize that our cohort predominantly comprises samples of gestational Week 8. In general, the decidua is reported to contain a remarkably large percentage of immune cells (40–50%) (Ander *et al.*, 2019; Xu *et al.*, 2021). In literature, the immune fraction of the early *decidua basalis* is described to comprise on average 60–70% dNK cells, 10–30% macrophages, and 3–20% T cells (Ander *et al.*, 2019; Yang *et al.*, 2019; Xu *et al.*, 2021). In accordance, our data shows that among T cells, macrophages, and dNK cells, dNK cells are the most abundant local immune cell type across all areas. We further show that the decidual cell abundance also significantly differs within the different areas of the heterogeneous first-trimester decidua. Surprisingly, the numbers of dNK cells, T cells, and macrophages decline with the degree of invasion (weak invasion > strong invasion > remodeling lesion). An immune cell distribution pattern in relation to trophoblast abundance, in line with our observations, is reported by von Rango *et al.* (2001), who suggest that EVTs exert a paracrine influence on their environment.

Neutrophils have rarely been the research focus and seldom have been suggested to occur substantially in invaded decidua (Amsalem *et al.*, 2014; Krop *et al.*, 2022; Mihalic *et al.*, 2023; Vondra *et al.*, 2023). Only in very recent reviews, have neutrophils been suggested to a key role in the decidua (Burton *et al.*, 2025; Fell *et al.*, 2025). Neutrophils are the most abundant circulating, but very short-lived, leukocyte subtype. In our study, extravasal neutrophils are almost exclusively located in the heavily invaded areas, in particular within or in close proximity to the remodeling lesions—hence, almost exclusively near EVT-compromised decidual blood vessels. It is therefore tempting to speculate that these neutrophils are extravasated from the eroded blood vessels. For example, in sterile liver injury, it is described that neutrophils travel within the vasculature toward the injury site, in response to DAMPs and chemotactic signals (Oliveira-Costa *et al.*, 2022). While neutrophils have long been regarded primarily as destructive cells, with their tissue-damaging effects considered locally restricted (as one would also expect in the decidua), recent overviews emphasize the beneficial effects of neutrophils within tissues (Oliveira-Costa *et al.*, 2022; Fell *et al.*, 2025). Neutrophils indeed exhibit phenotypic and functional plasticity upon activation and/or tissue infiltration and could play an important role in angiogenesis, wound healing, T-cell suppression, tissue repair, and tissue remodeling in the decidua, as suggested for other settings (Köstlin *et al.*, 2014; Kang *et al.*, 2016; Nadkarni *et al.*, 2016; Hellebrekers *et al.*, 2018; Ng *et al.*, 2019; Phillipson and Kubes, 2019; Oliveira-Costa *et al.*, 2022). Our spatial transcriptomics data highlights that the remodeling lesion area is characterized by an expression pattern of genes involved in ECM remodeling, tissue repair, angiogenesis, and vascular regulation, which may derive in part from neutrophils and/or other cell types responding to the formation of the remodeling lesion. In addition, neutrophil depletion in allogenic mouse pregnancy leads to abnormal placenta development and reduced viable litters (Nadkarni *et al.*, 2016; Ostrand-Rosenberg *et al.*, 2017; Hebeda *et al.*, 2022), suggesting that neutrophils play a critical role in human pregnancy as well. A neutrophil imbalance could therefore contribute to complications such as defective placental development or pregnancy loss (Croxatto *et al*., 2016).

Of note, due to several technical limitations, e.g. the low RNA abundance of circulating and tissue neutrophils, neutrophils are often overlooked in RNA datasets (Salcher *et al.*, 2022). As a result, neutrophils are usually severely underrepresented or not detected in single-cell RNA-seq studies, hindering the analysis of this immune cell type in the herein-used single-cell RNA-seq dataset (Vento-Tormo *et al.*, 2018a) and, hence, assessment with our deconvolution approach. However, the remodeling lesion area in our spatial transcriptomics data displays a gene expression profile linked to immune response and neutrophil-related processes/markers; importantly, the presence of extravasal neutrophils in the remodeling lesions was clearly shown with IHC.

Interestingly, there is a drop in dNK cells, T cells, and macrophages in the *zona spongiosa* compared to the weakly invaded area. Therefore, these immune cells appear to accumulate along the invasive front. In comparison to the non-invaded *zona spongiosa*, the non-invaded *zona compacta* is enriched in CD56^+^ dNK cells, CD14^+^CD163^+^ macrophages, and CD3^+^CD8^−^ T cells. The *zona compacta* needs to be a tolerogenic immune microenvironment that ‘welcomes’ trophoblast invasion while still providing defense against infections. dNK cells are highly abundant in the first-trimester decidua, especially in the *zona compacta*, where EVT invasion starts (from the cell columns), and in the weakly invaded areas, where the invasive process continues (as an invasive front). dNK cells facilitate immunotolerance, modulate EVT invasion, and promote vascular remodeling (Liu *et al.*, 2021; Wei *et al.*, 2022; Moffett and Shreeve, 2023). As recently demonstrated, several cytokines, which are only released by dNK cells, support *in vitro* differentiation of EVTs into different subtypes, which highlights the importance of a balanced immunological interplay between dNK cells and EVTs in successful placentation (Li *et al.*, 2024). Even in remodeling lesions, a substantial amount of dNK cells was detected, probably due to their important role in modulating EVT invasion and promoting remodeling of blood vessels (Liu *et al.*, 2021; Wei *et al.*, 2022; Moffett and Shreeve, 2023).

In this study, we combined classical histological approaches quantitatively assessed with semi-automated image analysis, integrative spatial and single-cell transcriptomics analysis, observer-based systematic evaluation for remodeling lesion-associated parameters, and staining series on consecutive serial decidua tissue sections using a comprehensive marker set. To investigate remodeling lesions and changes in relation to the degree of EVT invasion, we leveraged a key strength of our study—a large, well-characterized cohort of first-trimester human decidual tissues. Our cohort included matched *decidua basalis* and *parietalis* samples from the same donors, together with unique archival specimens. Of note, studies relying on first-trimester placental tissues from elective terminations are inherently limited, as surgical vacuum aspiration disrupts the intact (*in toto*) anatomical architecture of the decidua, and the eventual pregnancy outcome cannot be determined. With regard to the latter, it would be interesting to relate our observations to recurrent pregnancy loss or to study the impact of anticoagulation therapy on decidual remodeling lesions, which represent important avenues for further investigation. A limitation of this study may also be the lack of immune cell-depleted animal models. Of note, human placentation and placental anatomy differ fundamentally from those of commonly used animal models such as rodents, particularly regarding trophoblast invasion depth, spiral artery remodeling, and the composition and organization of the decidual immune compartment (Carter, 2021, 2024). Because of these substantial interspecies differences, such models cannot reliably recapitulate the human remodeling processes described in our study. While advanced *in vitro* models, including endometrial organoids, are emerging as valuable tools, they cannot fully recapitulate the complex immunovascular environment of the early *decidua basalis*. Such *in vitro* systems lack key physiological features, including maternal blood flow, hemodynamic forces, vascular permeability, and the continuous recruitment and extravasation of maternal immune cells. In addition, the limited *in vitro* lifespan of certain immune populations, particularly granulocytes, is substantially shorter than the time required for decidual and vascular remodeling, potentially precluding full assessment of these dynamic processes. Notwithstanding these limitations, our comprehensive approach based on our well-characterized human tissue cohort provides novel insights into the structural and cellular organization of the decidua during early human placental development.

Overall, we propose the concept that EVT invasion induces remodeling lesions in the *decidua basalis* and shapes the immune cell landscape of the early fetal–maternal interface. We characterize these remodeling lesions as focal areas in the decidua with strong EVT invasion, compromised tissue integrity, eroded vessels, and fibrin deposits. In addition, lower T cell, macrophage, and dNK cell abundance, and pronounced neutrophil niches in the perivascular decidual stroma, are associated with remodeling lesions. We believe that the occurrence of these remodeling lesions significantly influences the formation of placental fibrinoid layers, thereby contributing to the establishment of a stable yet flexible basal plate—and, in turn, to a reliable connection between mother and fetus. It can be speculated that inadequate decidual tissue restructuring and vascular adaptation lead to pregnancy pathologies and complications such as placental abruption or placenta accreta spectrum, as certain conditions can be associated with abnormally distributed fibrin deposits (Benirschke *et al.*, 2012; Jauniaux *et al.*, 2022). Taken together, we provide a new piece in the puzzle of tissue restructuring and vascular adaptation in early placental development. Future studies should define the temporal progression of remodeling lesions from EVT-mediated initiation to fully completed remodeling and provide a detailed characterization of the immune cell environment during this process, alongside the molecular profile of these lesions. Moreover, future studies should assess their occurrence in pregnancy pathologies and, with regard to clinical relevance, evaluate the impact of anticoagulation on decidual remodeling.

## Supplementary Material

deag078_Supplementary_Figure_S1

deag078_Supplementary_Figure_S2

deag078_Supplementary_Figure_S3

deag078_Supplementary_Figure_S4

deag078_Supplementary_Figure_S5

deag078_Supplementary_Figure_S6

deag078_Supplementary_Figure_S7

deag078_Supplementary_Figure_S8

deag078_Supplementary_Figure_S9

deag078_Supplementary_Figure_S10

deag078_Supplementary_Figure_S11

deag078_Supplementary_Figure_S12

deag078_Supplementary_Figure_S13

deag078_Supplementary_Figure_S14

deag078_Supplementary_Figure_S15

deag078_Supplementary_Figure_S16

deag078_Supplementary_Figure_S17

deag078_Supplementary_Figure_S18

deag078_Supplementary_Table_S1

deag078_Supplementary_Table_S2

deag078_Supplementary_Table_S3

## Data Availability

The data underlying this article are available in Gene Expression Omnibus repository at https://www.ncbi.nlm.nih.gov/geo/ and can be accessed with GSE301306.

## References

[deag078-B1] Amsalem H , Kwan M, Hazan A, Zhang J, Jones RL, Whittle W, Kingdom JCP, Croy BA, Lye SJ, Dunk CE. Identification of a novel neutrophil population: proangiogenic granulocytes in second-trimester human decidua. J Immunol 2014;193:3070–3079. 25135830 10.4049/jimmunol.1303117

[deag078-B2] Ander SE , Diamond MS, Coyne CB. Immune responses at the maternal-fetal interface. Sci Immunol 2019;4:eaat6114. 30635356 10.1126/sciimmunol.aat6114 PMC6744611

[deag078-B3] Arutyunyan A , Roberts K, Troulé K, Wong FCK, Sheridan MA, Kats I, Garcia-Alonso L, Velten B, Hoo R, Ruiz-Morales ER et al Spatial multiomics map of trophoblast development in early pregnancy. Nature 2023;616:143–151. 36991123 10.1038/s41586-023-05869-0 PMC10076224

[deag078-B4] Benirschke K , Burton GJ, Baergen RN. Pathology of the Human Placenta, 6th edn. Berlin, Heidelberg, Germany: Springer Berlin Heidelberg, 2012.

[deag078-B5] Burton GJ , Jauniaux E. The human placenta: new perspectives on its formation and function during early pregnancy. Proc Biol Sci 2023;290:20230191. 37072047 10.1098/rspb.2023.0191 PMC10113033

[deag078-B6] Burton GJ , Jauniaux E, Moffett A. Formation of the placental membranes and pathophysiological origin of associated great obstetrical syndromes. Am J Obstet Gynecol 2025;233:517–529. 40744286 10.1016/j.ajog.2025.07.039

[deag078-B7] Cable DM , Murray E, Zou LS, Goeva A, Macosko EZ, Chen F, Irizarry RA. Robust decomposition of cell type mixtures in spatial transcriptomics. Nat Biotechnol 2022;40:517–526. 33603203 10.1038/s41587-021-00830-w PMC8606190

[deag078-B8] Carter AM. Unique aspects of human placentation. Int J Mol Sci 2021;22:8099. 34360862 10.3390/ijms22158099 PMC8347521

[deag078-B9] Carter AM. Genomics, the diversification of mammals, and the evolution of placentation. Dev Biol 2024;516:167–182. 39173812 10.1016/j.ydbio.2024.08.011

[deag078-B10] Craven CM , Chedwick LR, Ward K. Placental basal plate formation is associated with fibrin deposition in decidual veins at sites of trophoblast cell invasion. Am J Obstet Gynecol 2002;186:291–296. 11854653 10.1067/mob.2002.119717

[deag078-B1990713] Croxatto D, , Micheletti A, , Montaldo E, , Orecchia P, , Loiacono F, , Canegallo F, , Calzetti F, , Fulcheri E, , Munari E, , Zamò A et al Group 3 innate lymphoid cells regulate neutrophil migration and function in human decidua. Mucosal Immunol 2016;9:1372–1383. 26906405 10.1038/mi.2016.10

[deag078-B11] Fell SL , Nemphos SM, Prusak JE, Kaur A, Lo JO, Manuzak JA. Neutrophils at the maternal-fetal interface: agents of protection or destruction? Am J Reprod Immunol 2025;94:e70181. 41165300 10.1111/aji.70181 PMC12574209

[deag078-B12] Fuchs J , Nonn O, Daxboeck C, Groiss S, Moser G, Gauster M, Lang-Olip I, Brislinger D. Automated quantitative image evaluation of antigen retrieval methods for 17 antibodies in placentation and implantation diagnostic and research. Microsc Microanal 2021;27:1–1517. 34851247 10.1017/S1431927621012630

[deag078-B13] Gorsek Sparovec T , Markert UR, Reif P, Schoell W, Moser G, Feichtinger J, Mihalic ZN, Kargl J, Gargett CE, Gold D. The fate of human SUSD2+ endometrial mesenchymal stem cells during decidualization. Stem Cell Res 2022;60:102671. 35093718 10.1016/j.scr.2022.102671

[deag078-B14] Gowhari Shabgah A , Jadidi-Niaragh F, Mohammadi H, Ebrahimzadeh F, Oveisee M, Jahanara A, Gholizadeh Navashenaq J. The role of atypical chemokine receptor D6 (ACKR2) in physiological and pathological conditions; friend, foe, or both? Front Immunol 2022;13:861931. 35677043 10.3389/fimmu.2022.861931 PMC9168005

[deag078-B15] Greenbaum S , Averbukh I, Soon E, Rizzuto G, Baranski A, Greenwald NF, Kagel A, Bosse M, Jaswa EG, Khair Z et al A spatially resolved timeline of the human maternal–fetal interface. Nature 2023;619:595–605. 37468587 10.1038/s41586-023-06298-9 PMC10356615

[deag078-B16] Guettler J , Forstner D, Cvirn G, Maninger S, Brugger BA, Nonn O, Kupper N, Pritz E, Wernitznig S, Dohr G et al Maternal platelets pass interstices of trophoblast columns and are not activated by HLA-G in early human pregnancy. J Reprod Immunol 2021;144:103280. 33530024 10.1016/j.jri.2021.103280

[deag078-B17] Hao Y , Hao S, Andersen-Nissen E, Mauck WM, Zheng S, Butler A, Lee MJ, Wilk AJ, Darby C, Zager M et al Integrated analysis of multimodal single-cell data. Cell 2021;184:3573–3587.e29. 34062119 10.1016/j.cell.2021.04.048 PMC8238499

[deag078-B18] Hao Y , Stuart T, Kowalski MH, Choudhary S, Hoffman P, Hartman A, Srivastava A, Molla G, Madad S, Fernandez-Granda C et al Dictionary learning for integrative, multimodal and scalable single-cell analysis. Nat Biotechnol 2024;42:293–304. 37231261 10.1038/s41587-023-01767-y PMC10928517

[deag078-B19] He N , van Iperen L, de Jong D, Szuhai K, Helmerhorst FM, van der Westerlaken LAJ, Chuva de Sousa Lopes SM. Human extravillous trophoblasts penetrate decidual veins and lymphatics before remodeling spiral arteries during early pregnancy. PLoS One 2017;12:e0169849. 28081266 10.1371/journal.pone.0169849 PMC5230788

[deag078-B20] Hebeda CB , Savioli AC, Scharf P, Paula-Silva MD, Gil CD, Farsky SHP, Sandri S. Neutrophil depletion in the pre-implantation phase impairs pregnancy index, placenta and fetus development. Front Immunol 2022;13:969336. 36248911 10.3389/fimmu.2022.969336 PMC9558710

[deag078-B21] Helige C , Ahammer H, Moser G, Hammer A, Dohr G, Huppertz B, Sedlmayr P. Distribution of decidual natural killer cells and macrophages in the neighbourhood of the trophoblast invasion front: a quantitative evaluation. Hum Reprod 2014;29:8–17. 24140594 10.1093/humrep/det353

[deag078-B22] Hellebrekers P , Vrisekoop N, Koenderman L. Neutrophil phenotypes in health and disease. Eur J Clin Invest 2018;48 Suppl 2:e12943. 29682724 10.1111/eci.12943 PMC6282827

[deag078-B23] Humar R , Schaer DJ, Vallelian F. Erythrophagocytes in hemolytic anemia, wound healing, and cancer. Trends Mol Med 2022;28:906–915. 36096988 10.1016/j.molmed.2022.08.005

[deag078-B24] Jauniaux E , Jurkovic D, Hussein AM, Burton GJ. New insights into the etiopathology of placenta accreta spectrum. Am J Obstet Gynecol 2022;227:384–391. 35248577 10.1016/j.ajog.2022.02.038

[deag078-B25] Kam EPY , Gardner L, Loke YW, King A. The role of trophoblast in the physiological change in decidual spiral arteries. Hum Reprod 1999;14:2131–2138. 10438439 10.1093/humrep/14.8.2131

[deag078-B26] Kang X , Zhang X, Liu Z, Xu H, Wang T, He L, Zhao A. Granulocytic myeloid-derived suppressor cells maintain feto-maternal tolerance by inducing Foxp3 expression in CD4+CD25-T cells by activation of the TGF-β/β-catenin pathway. Mol Hum Reprod 2016;22:499–511. 27016139 10.1093/molehr/gaw026

[deag078-B27] Kaufmann P , Huppertz B, Frank H-G. The fibrinoids of the human placenta: origin, composition and functional relevance. Ann Anat 1996;178:485–501. 9010564 10.1016/S0940-9602(96)80102-6

[deag078-B28] Korsunsky I , Millard N, Fan J, Slowikowski K, Zhang F, Wei K, Baglaenko Y, Brenner M, Loh P, Raychaudhuri S. Fast, sensitive and accurate integration of single-cell data with Harmony. Nat Methods 2019;16:1289–1296. 31740819 10.1038/s41592-019-0619-0 PMC6884693

[deag078-B29] Köstlin N , Kugel H, Spring B, Leiber A, Marmé A, Henes M, Rieber N, Hartl D, Poets CF, Gille C. Granulocytic myeloid derived suppressor cells expand in human pregnancy and modulate T‐cell responses. Eur J Immunol 2014;44:2582–2591. 24894988 10.1002/eji.201344200

[deag078-B30] Krop J , Zwan AVD, Ijsselsteijn ME, Kapsenberg H, Luk SJ, Hendriks SH, Keur CVD, Verleng LJ, Somarakis A, Meeren LVD et al Imaging mass cytometry reveals the prominent role of myeloid cells at the maternal-fetal interface. iScience 2022;25:104648. 35811852 10.1016/j.isci.2022.104648 PMC9257341

[deag078-B31] Krstic J , Deutsch A, Fuchs J, Gauster M, Gorsek Sparovec T, Hiden U, Krappinger JC, Moser G, Pansy K, Szmyra M et al (Dis)similarities between the decidual and tumor microenvironment. Biomedicines 2022;10:1065. 35625802 10.3390/biomedicines10051065 PMC9138511

[deag078-B32] Li Q , Sharkey A, Sheridan M, Magistrati E, Arutyunyan A, Huhn O, Sancho-Serra C, Anderson H, McGovern N, Esposito L et al Human uterine natural killer cells regulate differentiation of extravillous trophoblast early in pregnancy. Cell Stem Cell 2024;31:181–195.e9. 38237587 10.1016/j.stem.2023.12.013

[deag078-B33] Liu Y , Gao S, Zhao Y, Wang H, Pan Q, Shao Q. Decidual natural killer cells: a good nanny at the maternal-fetal interface during early pregnancy. Front Immunol 2021;12:663660. 34054831 10.3389/fimmu.2021.663660 PMC8149889

[deag078-B34] Luyendyk JP , Schoenecker JG, Flick MJ. The multifaceted role of fibrinogen in tissue injury and inflammation. Blood 2019;133:511–520. 30523120 10.1182/blood-2018-07-818211 PMC6367649

[deag078-B35] Lyssy F , Forstner D, Guettler J, Kupper N, Ujčič K, Neuper L, Daxboeck C, El-Heliebi A, Kummer D, Krappinger JC et al Maternal platelet-derived factors induce trophoblastic LAIR2 expression to promote trophoblast invasion and inhibit platelet activation at the fetal-maternal interface. J Thromb Haemost 2025;23:2010–2024. 40154792 10.1016/j.jtha.2025.03.020

[deag078-B36] Meekins JW , Luckas MJM, Pijnenborg R, McFadyen IR. Histological study of decidual spiral arteries and the presence of maternal erythrocytes in the intervillous space during the first trimester of normal human pregnancy. Placenta 1997;18:459–464. 9250710 10.1016/s0143-4004(97)80048-3

[deag078-B37] Mihalic ZN , Raftopoulou S, Kindler O, Sanchez AS, Maitz KS, Valadez-Cosmes P, Lackner A, Vondra S, Pollheimer J, Kargl J. POSTER 11 Neutrophil phenotype and function in first trimester pregnancy. J Reprod Immunol 2023;158:103594.

[deag078-B38] Moffett A , Shreeve N. Local immune recognition of trophoblast in early human pregnancy: controversies and questions. Nat Rev Immunol 2023;23:222–235. 36192648 10.1038/s41577-022-00777-2 PMC9527719

[deag078-B39] Mokhtar NM , Cheng C-W, Cook E, Bielby H, Smith SK, Charnock-Jones DS. Progestin regulates chemokine (C-X-C motif) ligand 14 transcript level in human endometrium. Mol Hum Reprod 2010;16:170–177. 19903701 10.1093/molehr/gap100

[deag078-B40] Moser G , Weiss G, Sundl M, Gauster M, Siwetz M, Lang-Olip I, Huppertz B. Extravillous trophoblasts invade more than uterine arteries: evidence for the invasion of uterine veins. Histochem Cell Biol 2017;147:353–366. 27774579 10.1007/s00418-016-1509-5 PMC5344955

[deag078-B41] Moser G , Windsperger K, Pollheimer J, Sousa Lopes SCd, Huppertz B. Human trophoblast invasion: new and unexpected routes and functions. Histochem Cell Biol 2018;150:361–370. 30046889 10.1007/s00418-018-1699-0 PMC6153604

[deag078-B42] Nadkarni S , Smith J, Sferruzzi-Perri AN, Ledwozyw A, Kishore M, Haas R, Mauro C, Williams DJ, Farsky SHP, Marelli-Berg FM, et al Neutrophils induce proangiogenic T cells with a regulatory phenotype in pregnancy. Proc Natl Acad Sci USA 2016;113:E8415–E8424. 27956610 10.1073/pnas.1611944114 PMC5206541

[deag078-B43] Ng LG , Ostuni R, Hidalgo A. Heterogeneity of neutrophils. Nat Rev Immunol 2019;19:255–265. 30816340 10.1038/s41577-019-0141-8

[deag078-B44] Nonn O , Debnath O, Valdes DS, Sallinger K, Secener AK, Fischer C, Tiesmeyer S, Nimo J, Kuenzer T, Ulrich J et al Senescent syncytiotrophoblast secretion during early onset preeclampsia. Hypertension 2025;82:787–799. 39440423 10.1161/HYPERTENSIONAHA.124.23362 PMC12002046

[deag078-B45] Oliveira-Costa KM , Menezes GB, Paula Neto HA. Neutrophil accumulation within tissues: a damage × healing dichotomy. Biomed Pharmacother 2022;145:112422. 34781139 10.1016/j.biopha.2021.112422

[deag078-B46] Ostrand-Rosenberg S , Sinha P, Figley C, Long R, Park D, Carter D, Clements VK. Frontline science: myeloid-derived suppressor cells (MDSCs) facilitate maternal–fetal tolerance in mice. J Leukoc Biol 2017;101:1091–1101. 28007981 10.1189/jlb.1HI1016-306RR PMC5380379

[deag078-B47] Patil I. Visualizations with statistical details: the “ggstatsplot” approach. JOSS 2021;6:3167.

[deag078-B48] Phillipson M , Kubes P. The healing power of neutrophils. Trends Immunol 2019;40:635–647. 31160208 10.1016/j.it.2019.05.001

[deag078-B49] Pijnenborg R , Vercruysse L, Hanssens M. The uterine spiral arteries in human pregnancy: facts and controversies. Placenta 2006;27:939–958. 16490251 10.1016/j.placenta.2005.12.006

[deag078-B50] Pollheimer J , Vondra S, Baltayeva J, Beristain AG, Knöfler M. Regulation of placental extravillous trophoblasts by the maternal uterine environment. Front Immunol 2018;9:2597. 30483261 10.3389/fimmu.2018.02597 PMC6243063

[deag078-B51] Robertson SA , Moldenhauer LM. Immunological determinants of implantation success. Int J Dev Biol 2014;58:205–217. 25023687 10.1387/ijdb.140096sr

[deag078-B52] Salcher S , Sturm G, Horvath L, Untergasser G, Kuempers C, Fotakis G, Panizzolo E, Martowicz A, Trebo M, Pall G et al High-resolution single-cell atlas reveals diversity and plasticity of tissue-resident neutrophils in non-small cell lung cancer. Cancer Cell 2022;40:1503–1520.e8. 36368318 10.1016/j.ccell.2022.10.008 PMC9767679

[deag078-B53] Suryawanshi H , Morozov P, Straus A, Sahasrabudhe N, Max KEA, Garzia A, Kustagi M, Tuschl T, Williams Z. A single-cell survey of the human first-trimester placenta and decidua. Sci Adv 2018;4:eaau4788. 30402542 10.1126/sciadv.aau4788 PMC6209386

[deag078-B54] Teoh PJ , Menzies FM, Hansell CAH, Clarke M, Waddell C, Burton GJ, Nelson SM, Nibbs RJB. Atypical chemokine receptor ACKR2 mediates chemokine scavenging by primary human trophoblasts and can regulate fetal growth, placental structure, and neonatal mortality in mice. J Immunol 2014;193:5218–5228. 25297873 10.4049/jimmunol.1401096

[deag078-B55] van der Zwan A , Unen VV, Beyrend G, Laban S, Keur CVD, Kapsenberg HJM, Höllt T, Chuva de Sousa Lopes SM, Hoorn M-LPvd, Koning F et al Visualizing dynamic changes at the maternal-fetal interface throughout human pregnancy by mass cytometry. Front Immunol 2020;11:571300. 33193353 10.3389/fimmu.2020.571300 PMC7649376

[deag078-B56] Vento-Tormo R , Efremova M, Botting RA, Turco MY, Vento-Tormo M, Meyer KB, Park J-E, Stephenson E, Polański K, Goncalves A et al Single-cell reconstruction of the early maternal–fetal interface in humans. Nature 2018a;563:347–353. 30429548 10.1038/s41586-018-0698-6 PMC7612850

[deag078-B57] Vento-Tormo R , Efremova M, Botting RA, Turco MY, Vento-Tormo M, Meyer KB, Park J-E, Stephenson E, Polański K, Goncalves A et al [dataset] *Reconstructing the Human First Trimester Fetal-Maternal Interface Using Single Cell Transcriptomics—10× data*. E-MTAB-6701: ArrayExpress, 2018b. https://www.ebi.ac.uk/biostudies/arrayexpress/studies/E-MTAB-6701 (Accessed 2020).

[deag078-B58] von Rango U , Classen-Linke I, Kertschanska S, Kemp B, Beier HM. Effects of trophoblast invasion on the distribution of leukocytes in uterine and tubal implantation sites. Fertil Steril 2001;76:116–124. 11438329 10.1016/s0015-0282(01)01859-3

[deag078-B59] von Rango U , Krusche CA, Kertschanska S, Alfer J, Kaufmann P, Beier HM. Apoptosis of extravillous trophoblast cells limits the trophoblast invasion in uterine but not in tubal pregnancy during first trimester. Placenta 2003;24:929–940. 14580375 10.1016/s0143-4004(03)00168-1

[deag078-B60] Vondra S , Höbler A-L, Lackner AI, Raffetseder J, Mihalic ZN, Vogel A, Saleh L, Kunihs V, Haslinger P, Wahrmann M et al The human placenta shapes the phenotype of decidual macrophages. Cell Rep 2023;42:111977. 36640334 10.1016/j.celrep.2022.111977

[deag078-B61] Wang Y , Lewis DF, Gu Y, Zhang Y, Alexander JS, Granger DN. Placental trophoblast-derived factors diminish endothelial barrier function. J Clin Endocrinol Metab 2004;89:2421–2428. 15126573 10.1210/jc.2003-031707

[deag078-B62] Wei X-W , Zhang Y-C, Wu F, Tian F-J, Lin Y. The role of extravillous trophoblasts and uterine NK cells in vascular remodeling during pregnancy. Front Immunol 2022;13:951482. 37408837 10.3389/fimmu.2022.951482 PMC10319396

[deag078-B63] Wickham H. *ggplot2*, 2nd edn. Cham, Germany: Springer International Publishing, 2016.

[deag078-B64] Windsperger K , Dekan S, Pils S, Golletz C, Kunihs V, Fiala C, Kristiansen G, Knöfler M, Pollheimer J. Extravillous trophoblast invasion of venous as well as lymphatic vessels is altered in idiopathic, recurrent, spontaneous abortions. Hum Reprod 2017;32:1208–1217. 28369440 10.1093/humrep/dex058

[deag078-B65] Xu L , Li Y, Sang Y, Li D-J, Du M. Crosstalk between trophoblasts and decidual immune cells: the cornerstone of maternal-fetal immunotolerance. Front Immunol 2021;12:642392. 33717198 10.3389/fimmu.2021.642392 PMC7947923

[deag078-B66] Yang F , Zheng Q, Jin L. Dynamic function and composition changes of immune cells during normal and pathological pregnancy at the maternal-fetal interface. Front Immunol 2019;10:2317. 31681264 10.3389/fimmu.2019.02317 PMC6813251

[deag078-B67] Yin Z , Su J, Lu L, Yang L, Su S, Jiang X. Visual identification of three kinds of human decidual tissues from elective termination of pregnancy. Placenta 2024;146:89–100. 38215630 10.1016/j.placenta.2024.01.006

