# Supplementary material for: Remodeling lesions locate at sites of strong extravillous trophoblast invasion and are associated with neutrophil presence in the human first-trimester decidua
Source: Hum Reprod. 2026 Jun 5;41(7):1078–96. doi: 10.1093/humrep/deag078 (PMC13334918; doi:10.1093/humrep/deag078)
Supplement: deag078_Supplementary_Figure_S1 [file deag078_supplementary_figure_s1.pdf]

Human Reproduction, 2026, 1–1
https://doi.org/10.1093/humrep/deag078
Supplementary Data

human reproduction

OXFORD

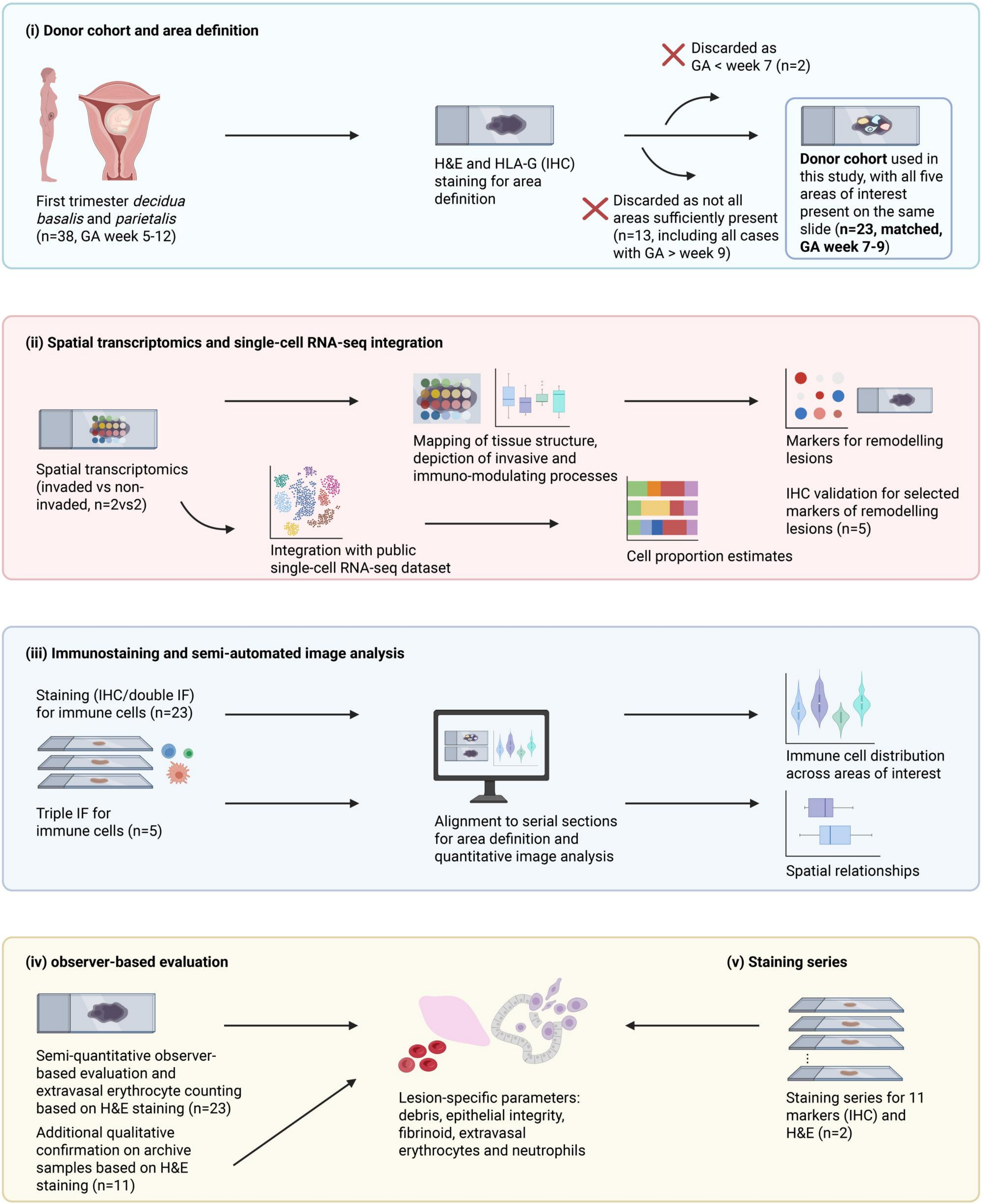

Supplementary Figure S1. **Workflow of this study.** First-trimester decidual tissue was thoroughly selected and categorized according to the degree of invasion to establish our donor cohort (n = 23). State-of-the-art spatial and single-cell transcriptomics analysis were combined with classical histological approaches and quantitative image assessment to systematically characterize the human first-trimester decidua in relation to the degree of EVT invasion—with a focus on the structural/morphological and cellular heterogeneity of the tissue. All analyses were performed on our donor cohort (either on all samples or on selected samples). Created in BioRender. Feichtinger, J. (2026) https://BioRender.com/jj7g7fz. GA, gestational age; IHC, immunohistochemistry; IF, immunofluorescence; H&E, hematoxylin and eosin.
