# Supplementary material for: Remodeling lesions locate at sites of strong extravillous trophoblast invasion and are associated with neutrophil presence in the human first-trimester decidua
Source: Hum Reprod. 2026 Jun 5;41(7):1078–96. doi: 10.1093/humrep/deag078 (PMC13334918; doi:10.1093/humrep/deag078)
Supplement: deag078_Supplementary_Figure_S2 [file deag078_supplementary_figure_s2.pdf]

Human Reproduction, 2026, 1–1
https://doi.org/10.1093/humrep/deag078
Supplementary Data

human reproduction

OXFORD

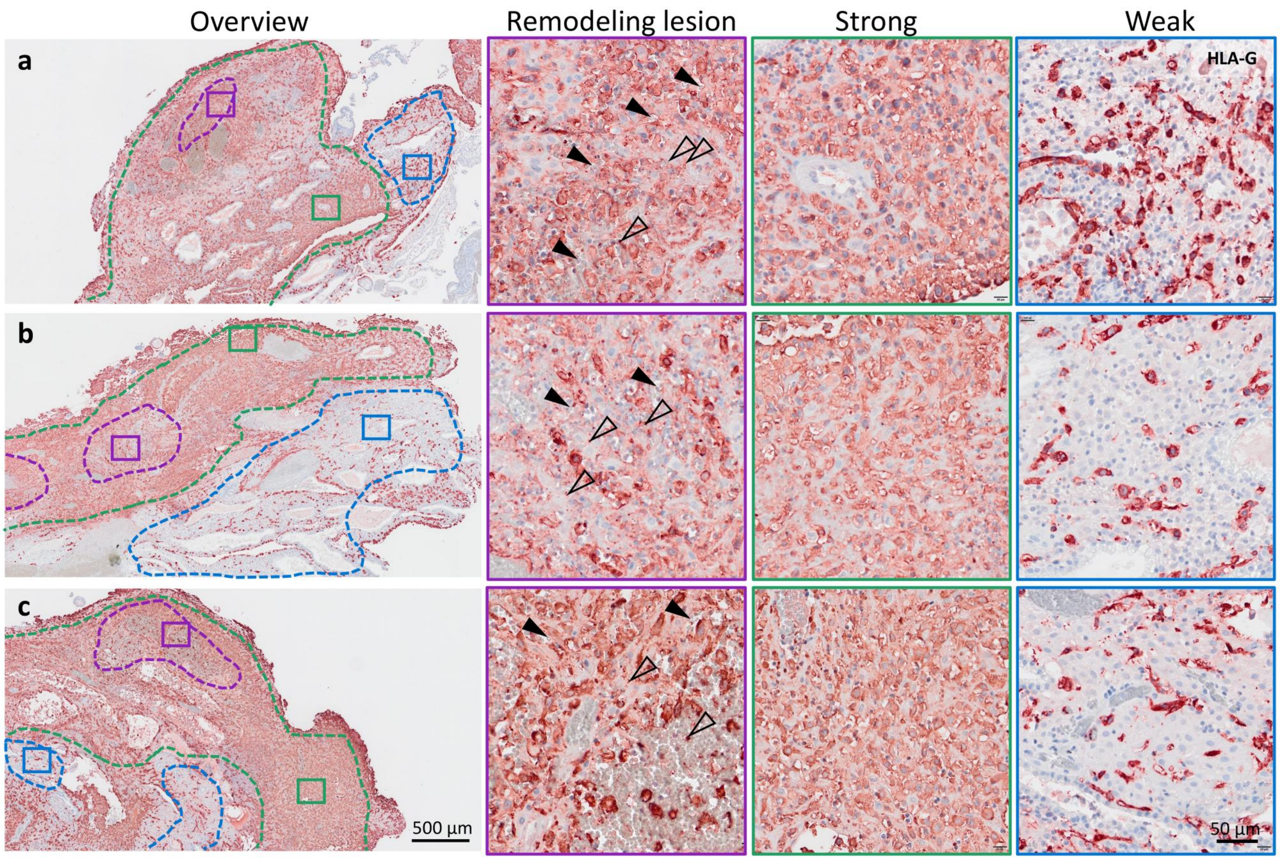

Supplementary Figure S2. Additional representative images of remodeling lesion areas as well as of strongly and weakly invaded areas in first-trimester *decidua basalis* immunostained for HLA-G, shown for three different donors of n = 23. (**a**–**c**) Remodeling lesion areas (dashed purple lines) are situated within strongly invaded areas (dashed green lines) and include signs of diminished tissue integrity, besides containing less intact nuclei, tatters of HLA-G that cannot be assigned to single cells (transparent arrowheads), and extravasal erythrocytes (black arrowheads)—as described in Table 1. Square insets show higher magnification of the remodeling lesions (within dashed purple line), strong (dashed green line), and weak (dashed blue line) invaded areas. Nuclear counterstain with hematoxylin.
