# Supplementary material for: Remodeling lesions locate at sites of strong extravillous trophoblast invasion and are associated with neutrophil presence in the human first-trimester decidua
Source: Hum Reprod. 2026 Jun 5;41(7):1078–96. doi: 10.1093/humrep/deag078 (PMC13334918; doi:10.1093/humrep/deag078)
Supplement: deag078_Supplementary_Figure_S3 [file deag078_supplementary_figure_s3.pdf]

Human Reproduction, 2026, 1–1
https://doi.org/10.1093/humrep/deag078
Supplementary Data

human reproduction
OXFORD

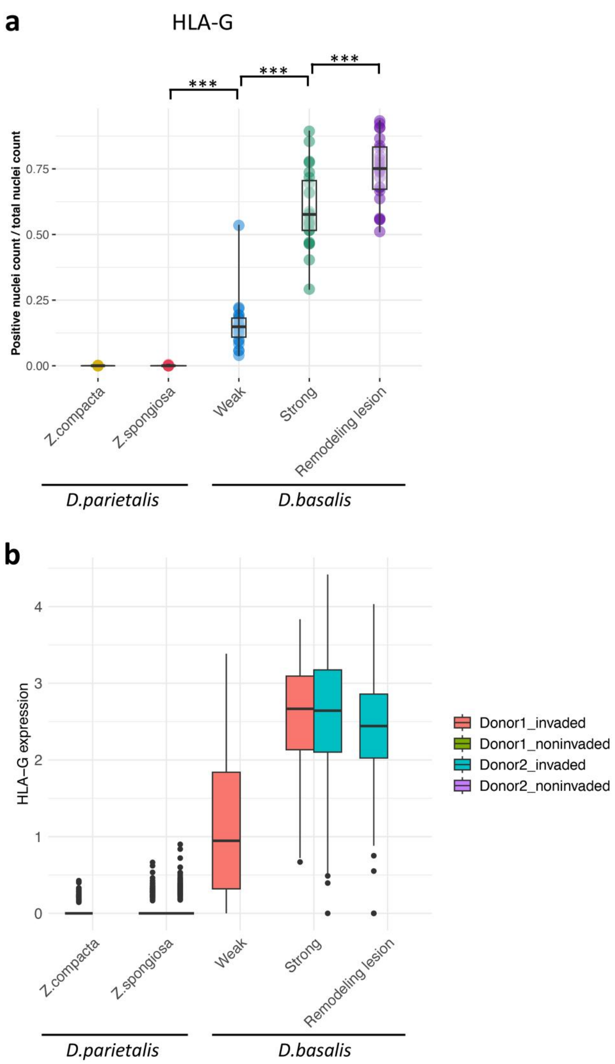

Supplementary Figure S3. Quantitative HLA-G measurements corroborate the tissue area definitions. (**a**) Extravillous trophoblast (EVT) cell abundance across the defined tissue regions (*decidua parietalis*: (i) *zona compacta*, (ii) *zona spongiosa*; *decidua basalis*: (iii) weak invasion, (iv) strong invasion, (v) remodeling lesion), quantitatively assessed in immunohistochemistry (IHC) images (n = 23) and visualized as number of HLA-G-positive nuclei per total nuclei. ***Adjusted *P*-value ≤ 0.001. To improve readability, only significant differences between adjacent groups are shown (results of all comparisons are listed in Supplementary Table S2). (**b**) Boxplots visualizing the level of normalized *HLA-G* expression of all spots assigned to the defined tissue areas annotated by histologists (see Fig. 2b; *decidua parietalis*: (i) *zona compacta*, (ii) *zona spongiosa*; *decidua basalis*: (iii) weak invasion, (iv) strong invasion, (v) remodeling lesion) for the four spatial transcriptomics capture areas. *Decidua basalis* and *parietalis* from two donors (Donor 1 and Donor 2, *decidua basalis* (invaded) and *decidua parietalis* (non-invaded)). D., decidua; Z., zona.
