# Supplementary material for: Remodeling lesions locate at sites of strong extravillous trophoblast invasion and are associated with neutrophil presence in the human first-trimester decidua
Source: Hum Reprod. 2026 Jun 5;41(7):1078–96. doi: 10.1093/humrep/deag078 (PMC13334918; doi:10.1093/humrep/deag078)
Supplement: deag078_Supplementary_Figure_S4 [file deag078_supplementary_figure_s4.pdf]

Human Reproduction, 2026, 1–1
https://doi.org/10.1093/humrep/deag078
Supplementary Data

human reproduction
OXFORD

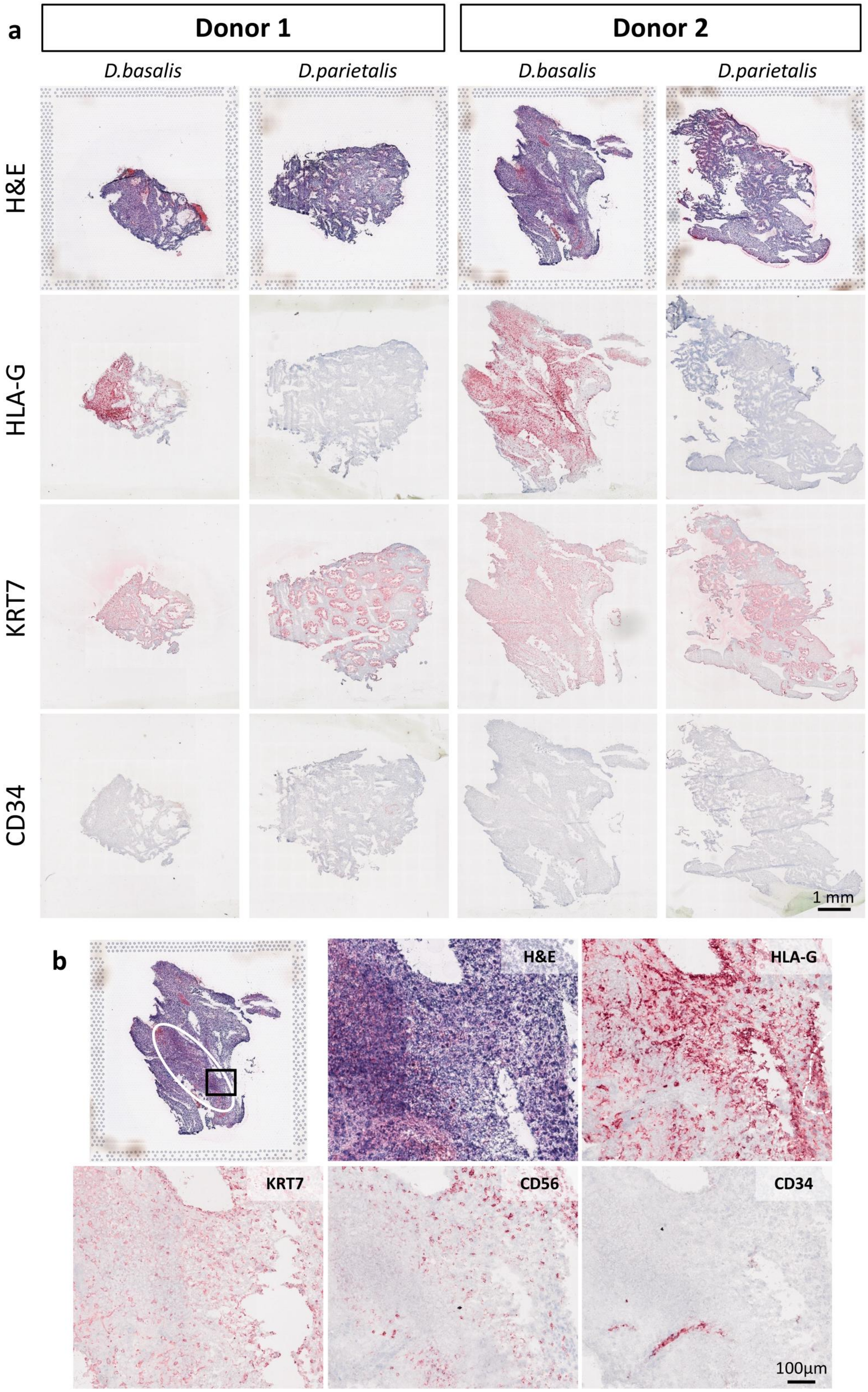

Supplementary Figure S4. Histological overview and characterization of the tissue mounted on the spatial transcriptomics gene expression slide. (**a**) *Decidua basalis* and *parietalis* from two donors (Donor 1, two left columns; Donor 2, two right columns) were cryosectioned (10 µm thick sections) and mounted on the spatial transcriptomics slide. After staining with hematoxylin and eosin (H&E), each capture area was imaged (Row 1) before processing for spatial transcriptomics analysis. Serial sections were immunostained for HLA-G, KRT7, and CD34 (Rows 2–4) to confirm sample selection. (**b**) A white eclipse highlights a remodeling lesion within the *decidua basalis* from Donor 2. Square inset shows an enlarged part of the remodeling lesion within immunostained serial sections (staining for HLA-G, KRT7, CD56, and CD34). Nuclear counterstain with hematoxylin. D., decidua.
