# Supplementary material for: Remodeling lesions locate at sites of strong extravillous trophoblast invasion and are associated with neutrophil presence in the human first-trimester decidua
Source: Hum Reprod. 2026 Jun 5;41(7):1078–96. doi: 10.1093/humrep/deag078 (PMC13334918; doi:10.1093/humrep/deag078)
Supplement: deag078_Supplementary_Figure_S5 [file deag078_supplementary_figure_s5.pdf]

Human Reproduction, 2026, 1–1
https://doi.org/10.1093/humrep/deag078
Supplementary Data

human reproduction

OXFORD

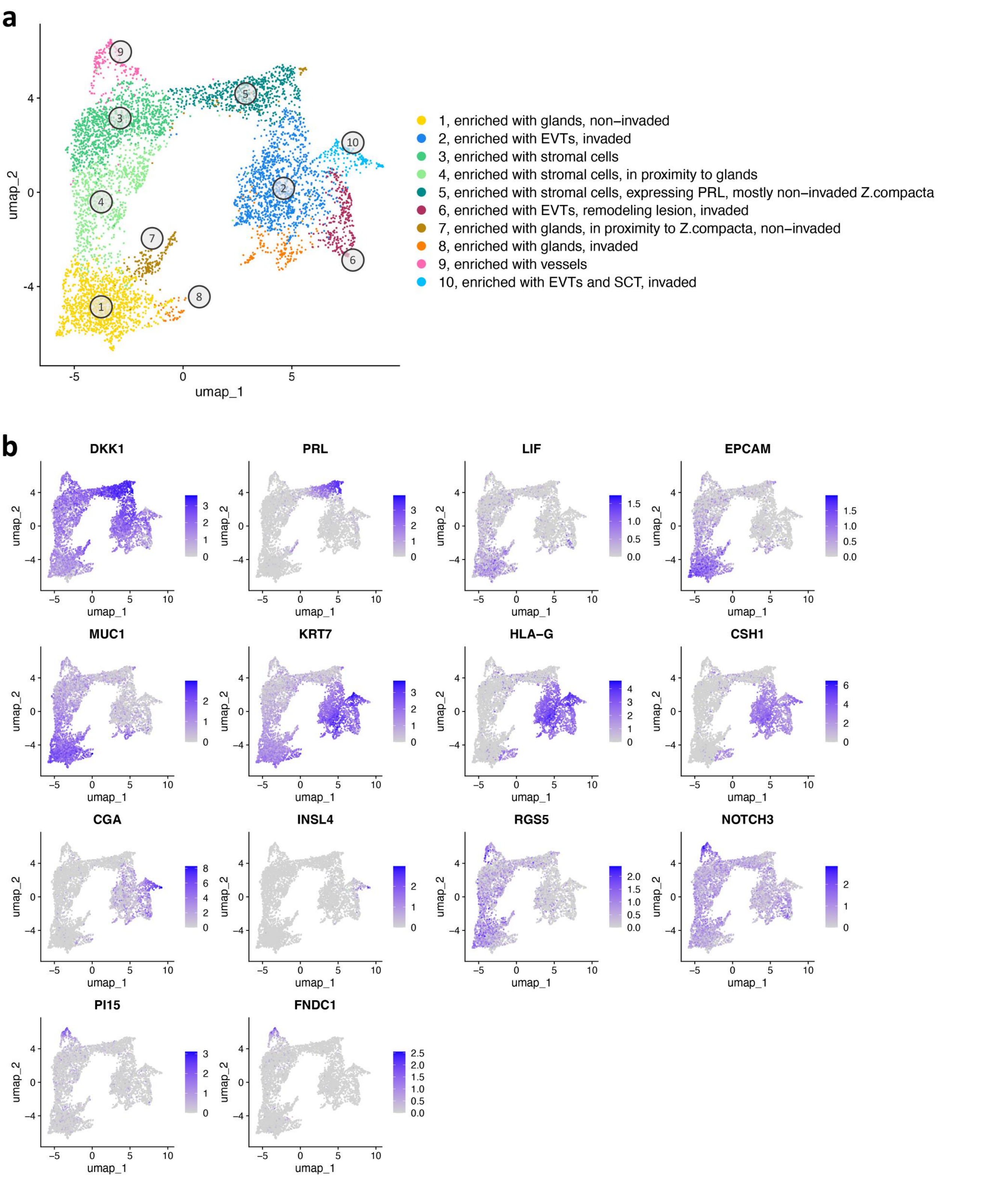

Supplementary Figure S5. Cluster annotation of the spatial transcriptomics data (*Decidua basalis* and *parietalis* from two donors). The clustering results were (**a**) visualized in Uniform Manifold Approximation and Projection (UMAP) space and annotated based on the corresponding hematoxylin and eosin (H&E) staining, *HLA-G* expression, and the expression of marker genes. The expression of selected marker genes was demonstrated with (**b**) feature plots. Single spots are colored based on the normalized expression level (shades from blue to gray encode a high to low value range). Z., zona; EVT, extravillous trophoblasts; SCT, syncytiotrophoblast.
