# Supplementary material for: Remodeling lesions locate at sites of strong extravillous trophoblast invasion and are associated with neutrophil presence in the human first-trimester decidua
Source: Hum Reprod. 2026 Jun 5;41(7):1078–96. doi: 10.1093/humrep/deag078 (PMC13334918; doi:10.1093/humrep/deag078)
Supplement: deag078_Supplementary_Figure_S6 [file deag078_supplementary_figure_s6.pdf]

Human Reproduction, 2026, 1–1
https://doi.org/10.1093/humrep/deag078
Supplementary Data

human reproduction

OXFORD

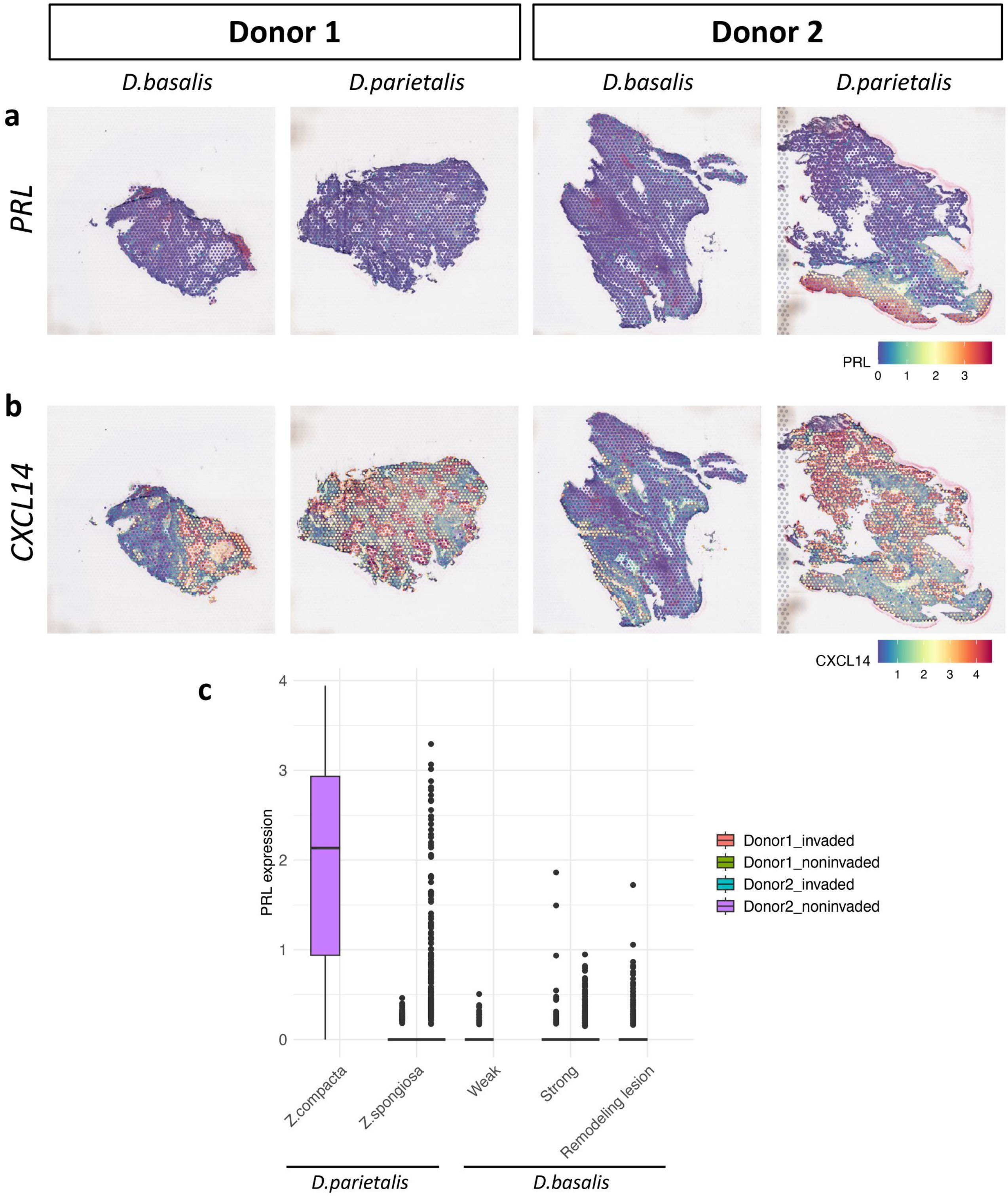

Supplementary Figure S6. Spatial expression pattern of selected genes. (**a**) *PRL* (encoding prolactin) is specifically expressed in the *zona compacta*. (**b**) The expression pattern of *CXCL14* particularly highlights the decidual glands. *Decidua basalis* and *parietalis* from two donors (normalized expression, shades from red to blue encode a high to low value range). (**c**) Boxplots visualizing the level of normalized *PRL* expression of all spots assigned to the defined tissue areas annotated by histologists (see Fig. 2b; *decidua parietalis*: (i) *zona compacta*, (ii) *zona spongiosa*; *decidua basalis*: (iii) weak invasion, (iv) strong invasion, (v) remodeling lesion) for the four spatial transcriptomics capture areas). D., decidua; Z., zona.
