# Supplementary material for: Remodeling lesions locate at sites of strong extravillous trophoblast invasion and are associated with neutrophil presence in the human first-trimester decidua
Source: Hum Reprod. 2026 Jun 5;41(7):1078–96. doi: 10.1093/humrep/deag078 (PMC13334918; doi:10.1093/humrep/deag078)
Supplement: deag078_Supplementary_Figure_S7 [file deag078_supplementary_figure_s7.pdf]

*Human Reproduction*, 2026, 1–1
https://doi.org/10.1093/humrep/deag078
**Supplementary Data**

human reproduction
OXFORD

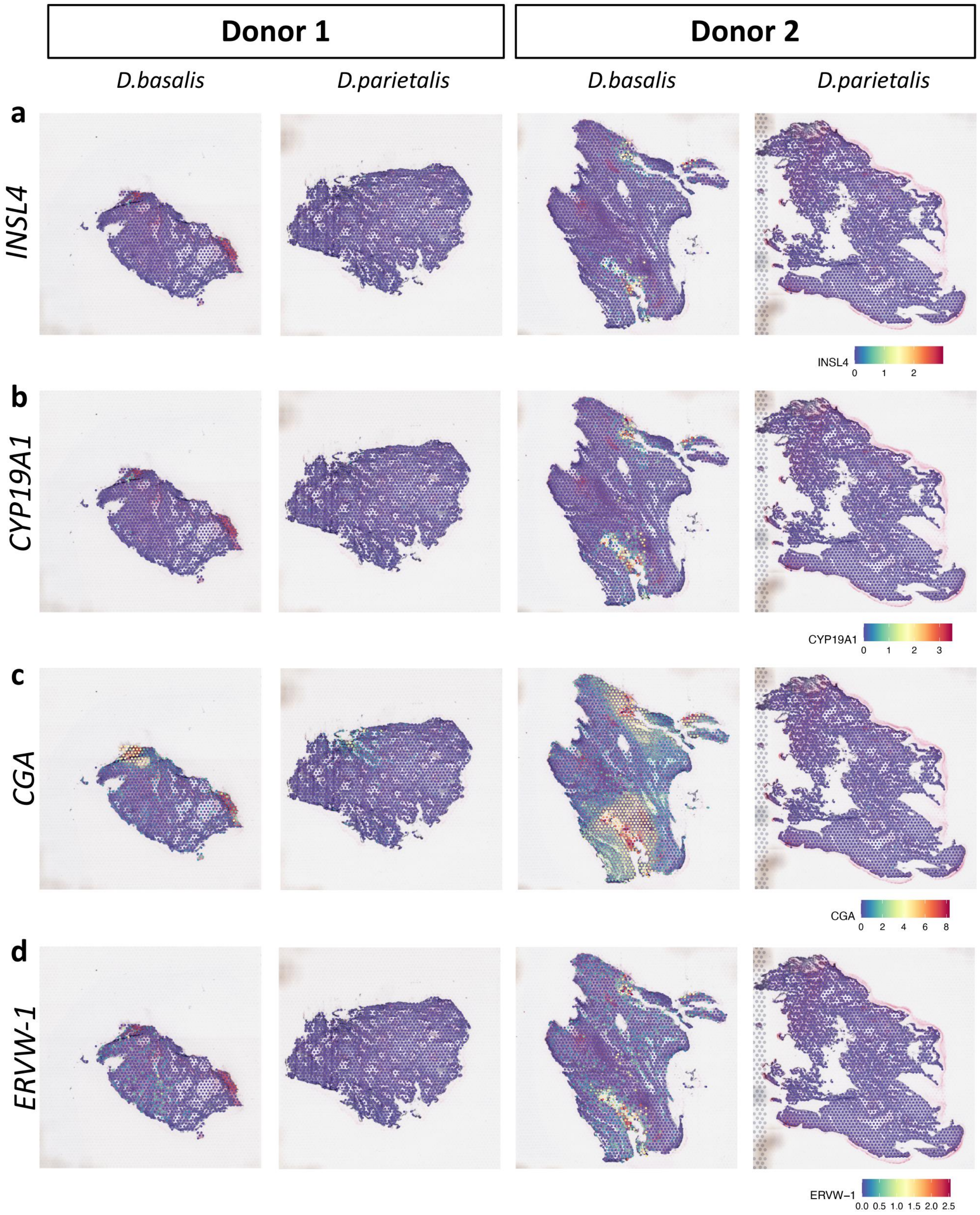


**Supplementary Figure S7.** **Spatial expression pattern of marker genes for the syncytiotrophoblast (SCT): (a) *INSL4*, (b) *CYP19A1*, (c) *CGA*, and (d) *ERVW-1*** (normalized expression, shades from red to blue encode a high to low value range). *Decidua basalis* and *parietalis* from two donors. D., decidua.
