# Supplementary material for: Remodeling lesions locate at sites of strong extravillous trophoblast invasion and are associated with neutrophil presence in the human first-trimester decidua
Source: Hum Reprod. 2026 Jun 5;41(7):1078–96. doi: 10.1093/humrep/deag078 (PMC13334918; doi:10.1093/humrep/deag078)
Supplement: deag078_Supplementary_Figure_S8 [file deag078_supplementary_figure_s8.pdf]

Human Reproduction, 2026, 1–1
https://doi.org/10.1093/humrep/deag078
Supplementary Data

human reproduction

OXFORD

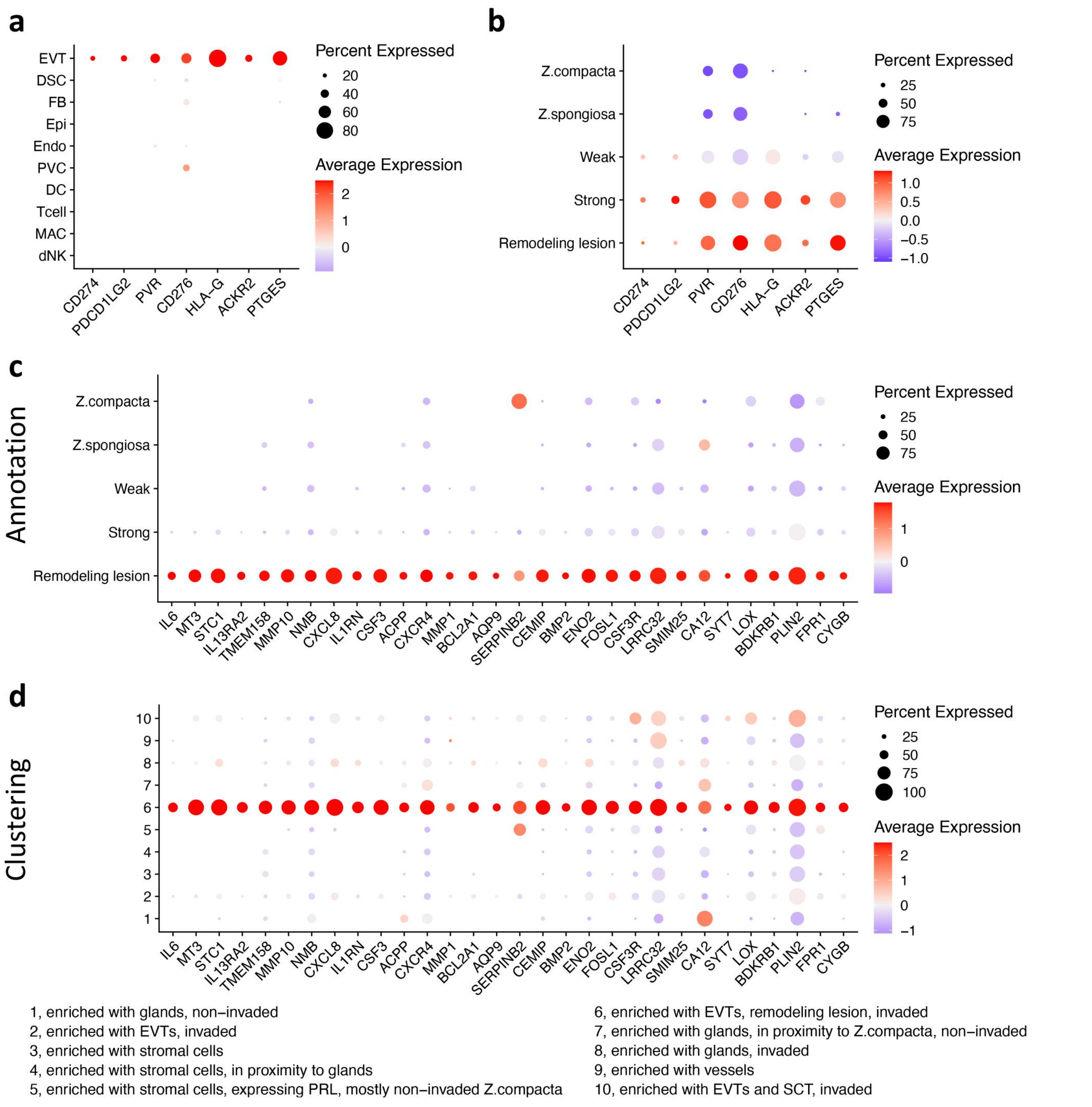

Supplementary Figure S8. Gene expression pattern of selected markers and immunomodulating molecules. (**a**) Expression of *CD274*, *PDCD1LG2*, *PVR*, *CD276*, *HLA-G*, *ACKR2*, and *PTGES* in (**a**) a publicly available first-trimester single-cell RNA-seq dataset (Vento-Tormo *et al.*, 2018a,b) and expression pattern of these markers (**b**) in our spatial transcriptomics dataset, annotated based on our defined areas (*decidua parietalis*: (i) *zona compacta*, (ii) *zona spongiosa*; *decidua basalis*: (iii) weak, (iv) strong, (v) remodeling lesion) and visualized as dot plot. *Decidua basalis* and *parietalis* from two donors. (**c**) Expression pattern of the top 30 upregulated genes between the remodeling lesion and strongly invaded areas (annotation based), visualized as dot plot. (**d**) Expression pattern of these 30 genes visualized for the cluster-based annotation, showing that these genes have a similar expression pattern in Cluster #6 (remodeling lesion) compared with the manual annotated remodeling lesion. The color indicates the average expression level (scaled normalized expression, shades from red to blue encode a high to low value range), while the dot size represents the percentage of cells expressing the gene. dNK, decidual natural killer cells; T cell, T cells; MAC, macrophages; DC, dendritic cells; DSC, decidual stromal cells, FB, decidual fibroblasts; Endo, endothelial cells; PVC, perivascular cells; Epi, epithelial cells; EVTs, extravillous trophoblasts; SCT, syncytiotrophoblast; *PRL*, gene encoding prolactin; Z., zona.
