# Supplementary material for: Remodeling lesions locate at sites of strong extravillous trophoblast invasion and are associated with neutrophil presence in the human first-trimester decidua
Source: Hum Reprod. 2026 Jun 5;41(7):1078–96. doi: 10.1093/humrep/deag078 (PMC13334918; doi:10.1093/humrep/deag078)
Supplement: deag078_Supplementary_Figure_S9 [file deag078_supplementary_figure_s9.pdf]

Human Reproduction, 2026, 1–1
https://doi.org/10.1093/humrep/deag078
Supplementary Data

human reproduction
OXFORD

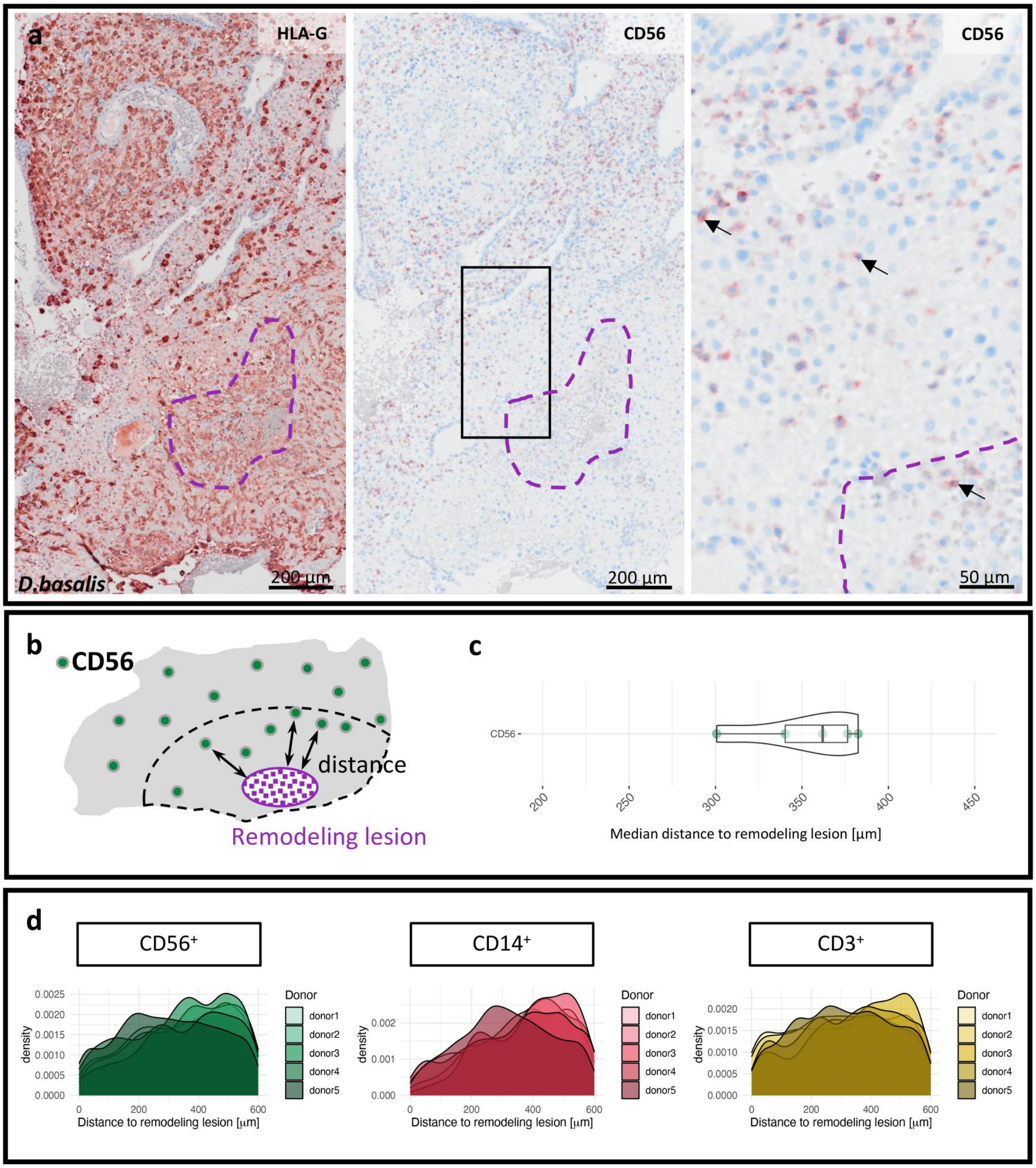

Supplementary Figure S9. Detection of main immune cell types in the *decidua basalis* and their spatial relationships, with a focus on their proximity to remodeling lesions. (**a**) Serial sections (n = 5) were assessed by immunohistochemistry to visualize extravillous trophoblast (EVT) invasion (HLA-G) and distribution of decidual natural killer (dNK) cells ($CD56^+$, black arrows). Within a remodeling lesion (in the *decidua basalis*, dashed purple line), dNK cells are present both directly in and at a greater distance from the remodeling lesions. Only few are directly adjacent to the remodeling lesions. Nuclear counterstain with hematoxylin. Black inset indicates enlargement shown in the third column. (**b**) Schematic showing the quantitative distance measurements of $CD56^+$ dNK cells to the nearest remodeling lesion within a defined measurement area (dashed black line). (**c**) Boxplot visualizing the median distance [µm] of $CD56^+$ dNK cells to the remodeling lesions. (**d**) Density plots visualize the spatial relationship of $CD56^+$ dNK cells, $CD3^+$ T cells, and $CD14^+$ macrophages to the remodeling lesions. The distance distribution profiles are visualized for each donor (n = 5) and for $CD56^+$ dNK cells (green), $CD14^+$ macrophages (red) and $CD3^+$ T cells (yellow), respectively. The distances of cells are measured to the nearest remodeling lesion in [µm]. D., decidua.
