# Supplementary material for: Remodeling lesions locate at sites of strong extravillous trophoblast invasion and are associated with neutrophil presence in the human first-trimester decidua
Source: Hum Reprod. 2026 Jun 5;41(7):1078–96. doi: 10.1093/humrep/deag078 (PMC13334918; doi:10.1093/humrep/deag078)
Supplement: deag078_Supplementary_Figure_S11 [file deag078_supplementary_figure_s11.pdf]

Human Reproduction, 2026, 1–1
https://doi.org/10.1093/humrep/deag078
Supplementary Data

human reproduction
OXFORD

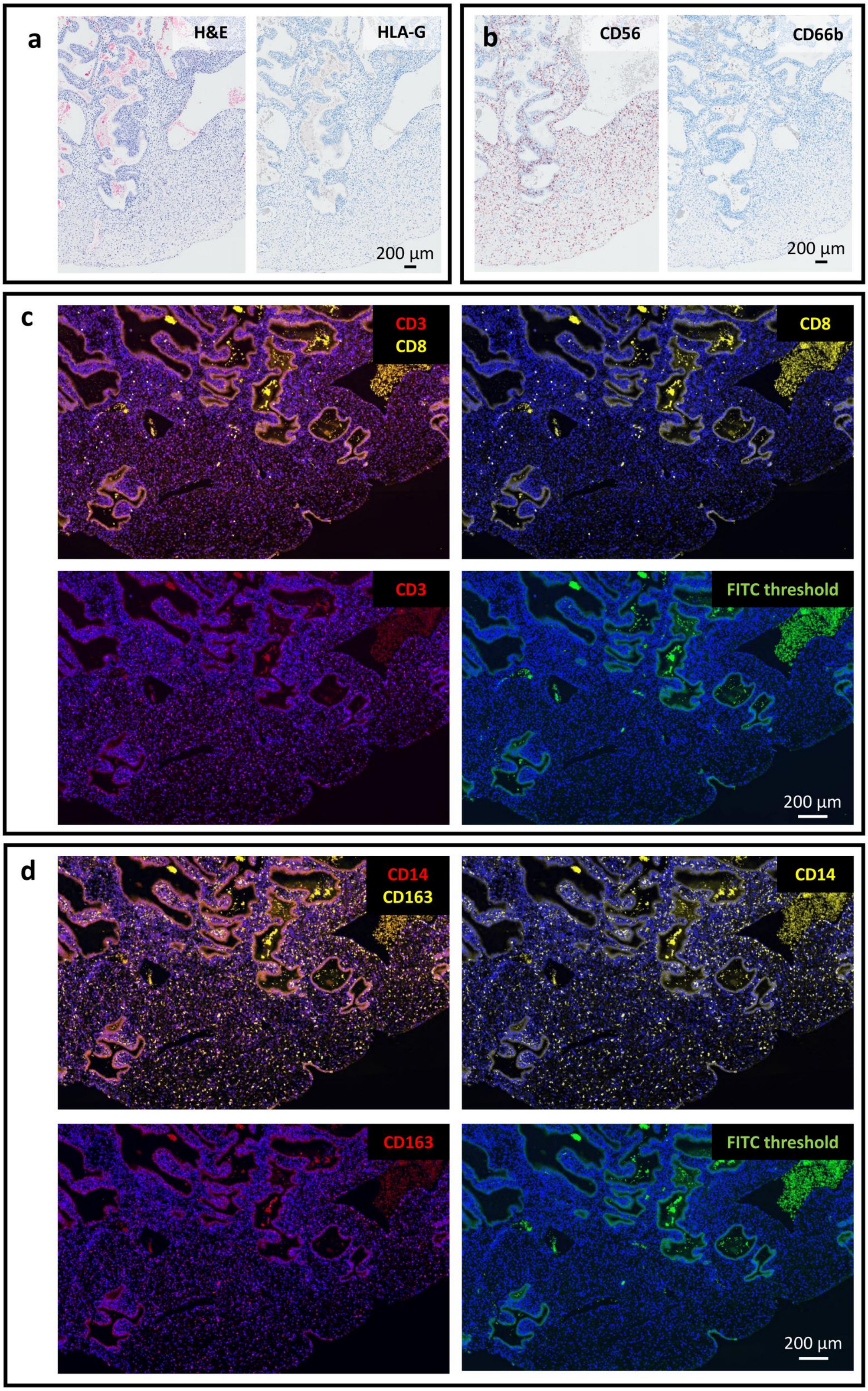

Supplementary Figure S11. Key immune cell populations in first-trimester *decidua parietalis*—representative images for area-specific image-based quantification of immune cells (exemplified shown for one donor of n = 23). Serial sections were assessed by immunohistochemistry (IHC) or double immunofluorescence (IF) to (**a**) visualize tissue morphology and EVT invasion (hematoxylin and eosin (H&E), HLA-G), (**b**) decidual natural killer (dNK) cells (CD56) and neutrophils (CD66b), (**c**) T-cells (CD3, CD8), and (**d**) macrophages (CD14, CD163). Nuclear counterstain with hematoxylin for IHC or DAPI for IF.
