# Supplementary material for: Remodeling lesions locate at sites of strong extravillous trophoblast invasion and are associated with neutrophil presence in the human first-trimester decidua
Source: Hum Reprod. 2026 Jun 5;41(7):1078–96. doi: 10.1093/humrep/deag078 (PMC13334918; doi:10.1093/humrep/deag078)
Supplement: deag078_Supplementary_Figure_S12 [file deag078_supplementary_figure_s12.pdf]

Human Reproduction, 2026, 1–1
https://doi.org/10.1093/humrep/deag078
Supplementary Data

human reproduction

OXFORD

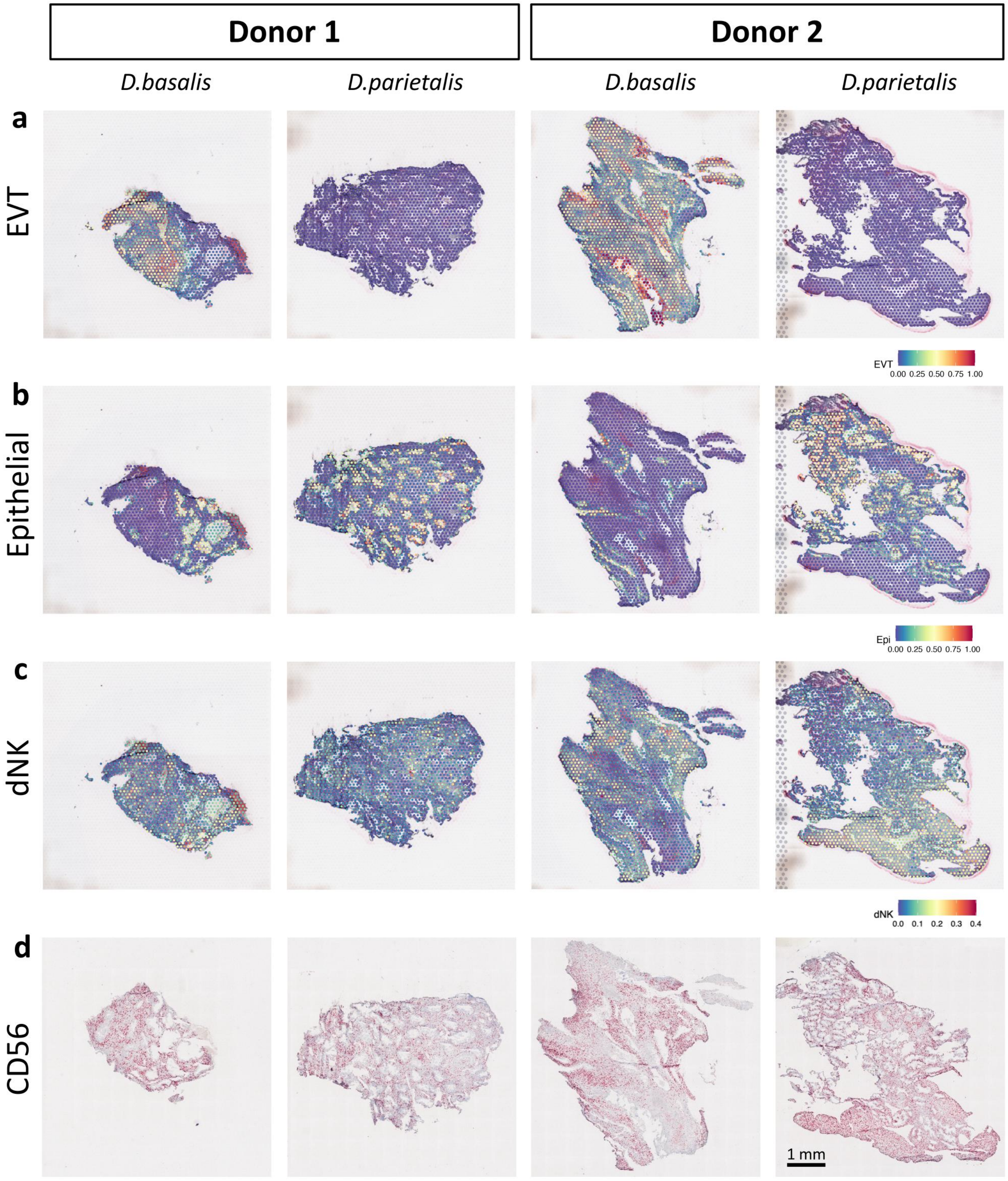

Supplementary Figure S12. Spatial distribution of estimated cell proportions for (a) extravillous trophoblasts (EVTs), (b) epithelial cells, and (c) decidual natural killer (dNK) cells, derived from the integration of our spatial transcriptomics and publicly available single-cell RNA-seq data. (**d**) Serial sections of decidua tissue mounted on the spatial transcriptomics gene expression slide were immunostained for CD56. *Decidua basalis* and *parietalis* from two donors. D., decidua.
