# Supplementary material for: Remodeling lesions locate at sites of strong extravillous trophoblast invasion and are associated with neutrophil presence in the human first-trimester decidua
Source: Hum Reprod. 2026 Jun 5;41(7):1078–96. doi: 10.1093/humrep/deag078 (PMC13334918; doi:10.1093/humrep/deag078)
Supplement: deag078_Supplementary_Figure_S13 [file deag078_supplementary_figure_s13.pdf]

Human Reproduction, 2026, 1–1
https://doi.org/10.1093/humrep/deag078
Supplementary Data

human reproduction
OXFORD

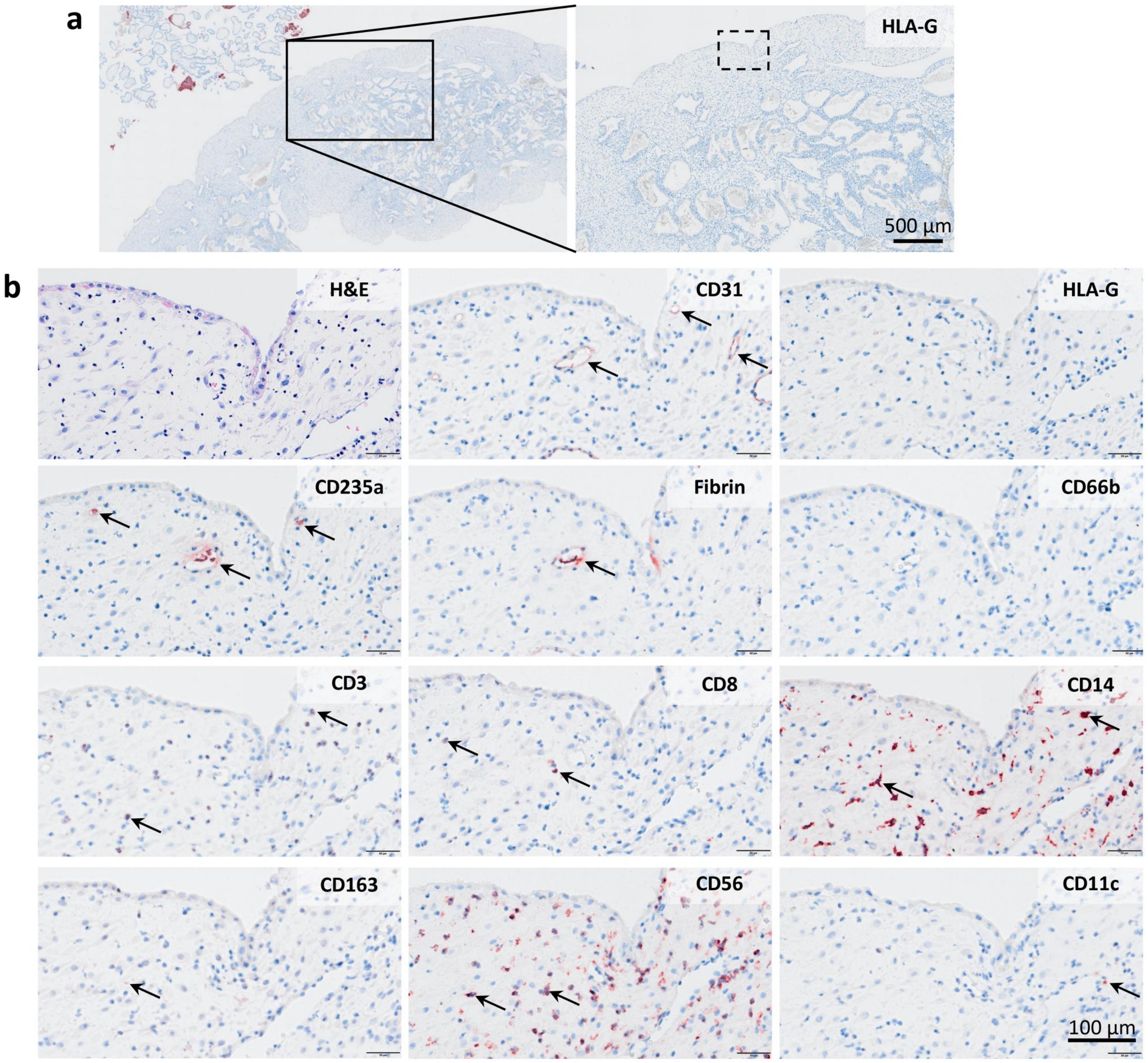

Supplementary Figure S13. Staining series of the non-invaded *decidua parietalis*. (**a**) Overview of the assessed *decidua parietalis* immunostained for HLA-G. Note that extravillous trophoblast (EVT) cell columns of placental villi in the upper right corner are HLA-G positive. Dashed black inset in the upper right image highlights an example region within the *zona compacta* presented in higher magnification in (**b**). Serial sections were stained with hematoxylin and eosin (H&E) and immunostained for CD31 (endothelium), HLA-G (EVTs), CD235a (erythrocytes), fibrin, CD66b (neutrophils), CD3 (T cells), CD8 (cytotoxic T cells), CD14 (macrophages), CD163 (macrophage differentiation marker), CD56 (natural killer (NK) cells), and CD11c (dendritic cells) (exemplified shown for one donor of n = 2). Arrows highlight the respective cell types or structures. Nuclear counterstain with hematoxylin. The *decidua parietalis* shows an intact, continuous epithelial surface. Underneath the epithelium, the densely packed *zona compacta* is found including vessels, very few glands, scattered T cells, macrophages, dNK cells, and dendritic cells within the tissue stroma, and is not invaded by EVTs. Vessels present with erythrocytes and at times with fibrin within the lumen, while their endothelium is intact. Neither extravasal neutrophils nor fibrin can be detected.
