# Supplementary material for: Remodeling lesions locate at sites of strong extravillous trophoblast invasion and are associated with neutrophil presence in the human first-trimester decidua
Source: Hum Reprod. 2026 Jun 5;41(7):1078–96. doi: 10.1093/humrep/deag078 (PMC13334918; doi:10.1093/humrep/deag078)
Supplement: deag078_Supplementary_Figure_S14 [file deag078_supplementary_figure_s14.pdf]

Human Reproduction, 2026, 1–1
https://doi.org/10.1093/humrep/deag078
Supplementary Data

human reproduction
OXFORD

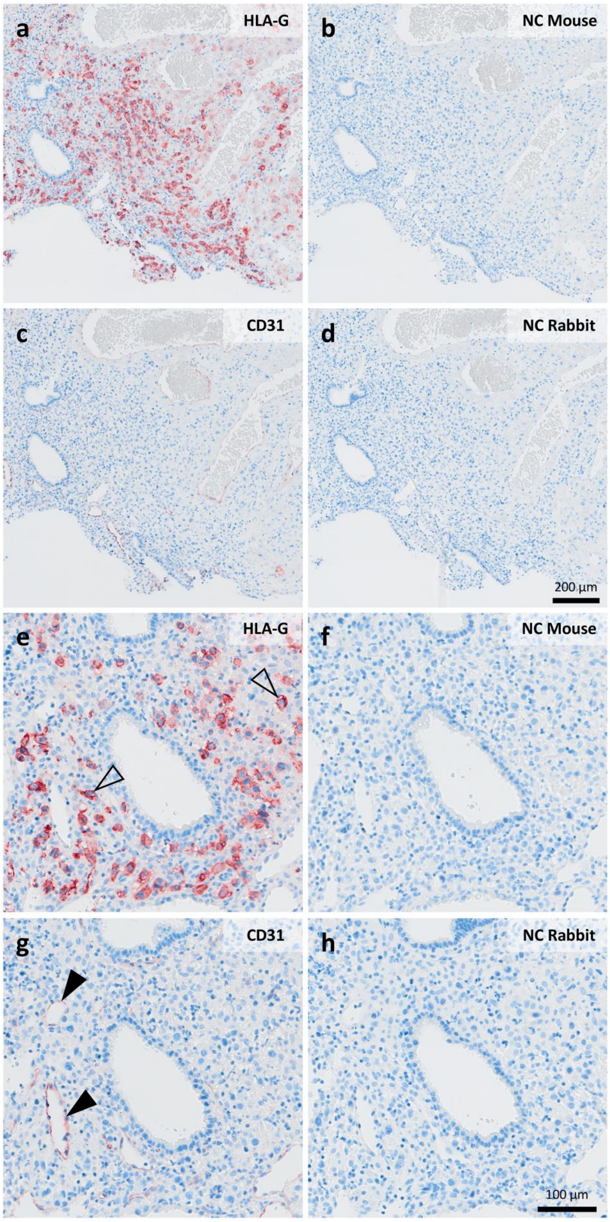

Supplementary Figure S14. Immunohistochemistry negative controls (NC) in lower (a–d) and higher magnification (e–h). Serial sections of human *decidua basalis* were stained with (a) mouse anti-HLA-G antibody, (b) negative control mouse immunoglobulin fraction, (c) rabbit anti-CD31 antibody, and (d) negative control rabbit immunoglobulin fraction. (e-h) HLA-G-positive cells are indicated with transparent arrowheads. Positive CD31 staining, representing the vascular endothelial lining, is indicated with black arrowheads. Negative control sections revealed the absence of specific staining, confirming antibody specificity. The scale bar in (d) is representative of images (a–d), and the scale bar in (h) is representative of images (e–h).
