# Supplementary material for: Remodeling lesions locate at sites of strong extravillous trophoblast invasion and are associated with neutrophil presence in the human first-trimester decidua
Source: Hum Reprod. 2026 Jun 5;41(7):1078–96. doi: 10.1093/humrep/deag078 (PMC13334918; doi:10.1093/humrep/deag078)
Supplement: deag078_Supplementary_Figure_S15 [file deag078_supplementary_figure_s15.pdf]

Human Reproduction, 2026, 1–1
https://doi.org/10.1093/humrep/deag078
Supplementary Data
human reproduction
OXFORD

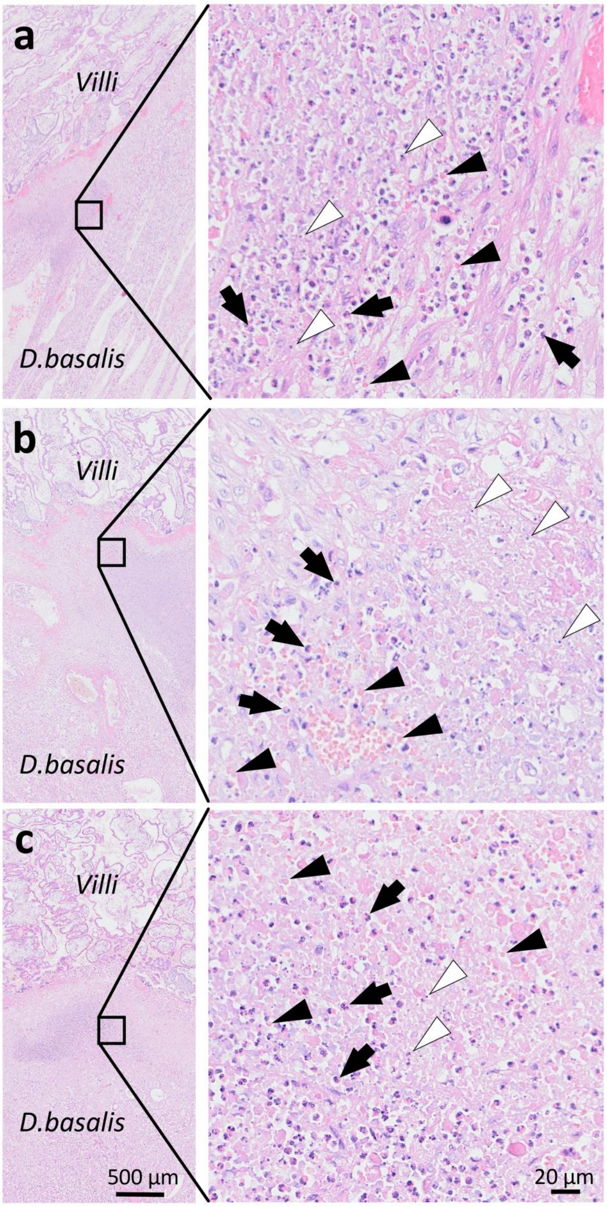

Supplementary Figure S15. Remodeling lesions in first-trimester *decidua basalis* in archival tissues obtained in previous decades, exemplified shown in samples from three different donors of n = 11 (a–c). Remodeling lesions in the hematoxylin and eosin (H&E)-stained sections are characterized by debris (white arrowheads), extravasal erythrocytes (black arrowheads), neutrophils (black arrows), and presence of fibrinoid.
