# Supplementary material for: Remodeling lesions locate at sites of strong extravillous trophoblast invasion and are associated with neutrophil presence in the human first-trimester decidua
Source: Hum Reprod. 2026 Jun 5;41(7):1078–96. doi: 10.1093/humrep/deag078 (PMC13334918; doi:10.1093/humrep/deag078)
Supplement: deag078_Supplementary_Figure_S16 [file deag078_supplementary_figure_s16.pdf]

Human Reproduction, 2026, 1–1
https://doi.org/10.1093/humrep/deag078
Supplementary Data

human reproduction
OXFORD

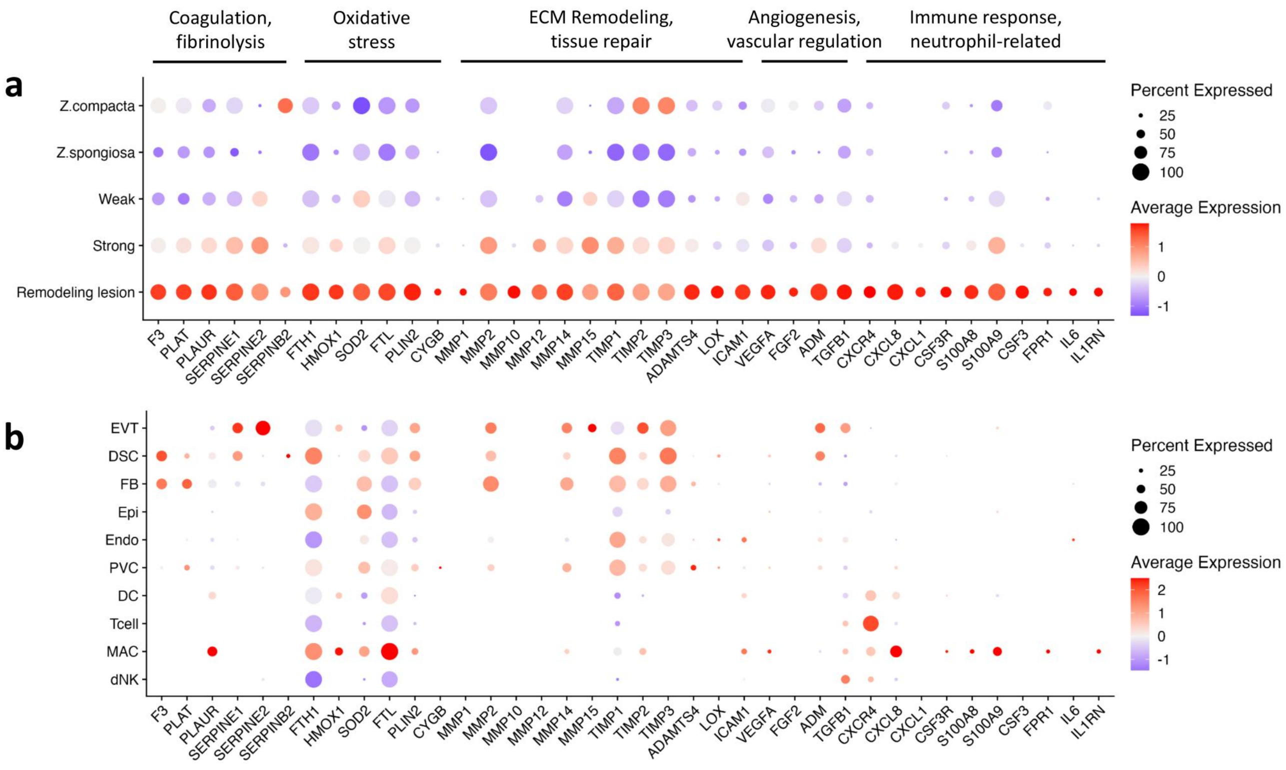

Supplementary Figure S16. Gene expression pattern of selected molecules related to coagulation, fibrinolysis, oxidative stress, extracellular matrix (ECM) remodeling, tissue repair, angiogenesis, vascular regulation, immune response, and neutrophil-related processes. Expression of these markers in (**a**) our spatial transcriptomics dataset, annotated based on our defined areas (*decidua parietalis*: (i) *zona compacta*, (ii) *zona spongiosa*; *decidua basalis*: (iii) weak invasion, (iv) strong invasion, (v) remodeling lesion; *decidua basalis* and *parietalis* from two donors), and in (**b**) a publicly available first-trimester single-cell RNA-seq dataset (Vento-Tormo *et al.*, 2018a,b), visualized as dot plot. The color indicates the average expression level (scaled normalized expression, shades from red to blue encode a high to low value range), while the dot size represents the percentage of cells expressing the gene. dNK, decidual natural killer cells; Tcell, T cells; MAC, macrophages; DC dendritic cells; DSC, decidual stromal cells, FB, decidual fibroblasts; Endo, endothelial cells; PVC, perivascular cells; Epi, epithelial cells; EVTs, extravillous trophoblasts; Z., zona.
