# Supplementary material for: Remodeling lesions locate at sites of strong extravillous trophoblast invasion and are associated with neutrophil presence in the human first-trimester decidua
Source: Hum Reprod. 2026 Jun 5;41(7):1078–96. doi: 10.1093/humrep/deag078 (PMC13334918; doi:10.1093/humrep/deag078)
Supplement: deag078_Supplementary_Figure_S17 [file deag078_supplementary_figure_s17.pdf]

Human Reproduction, 2026, 1–1
https://doi.org/10.1093/humrep/deag078
Supplementary Data

human reproduction
OXFORD

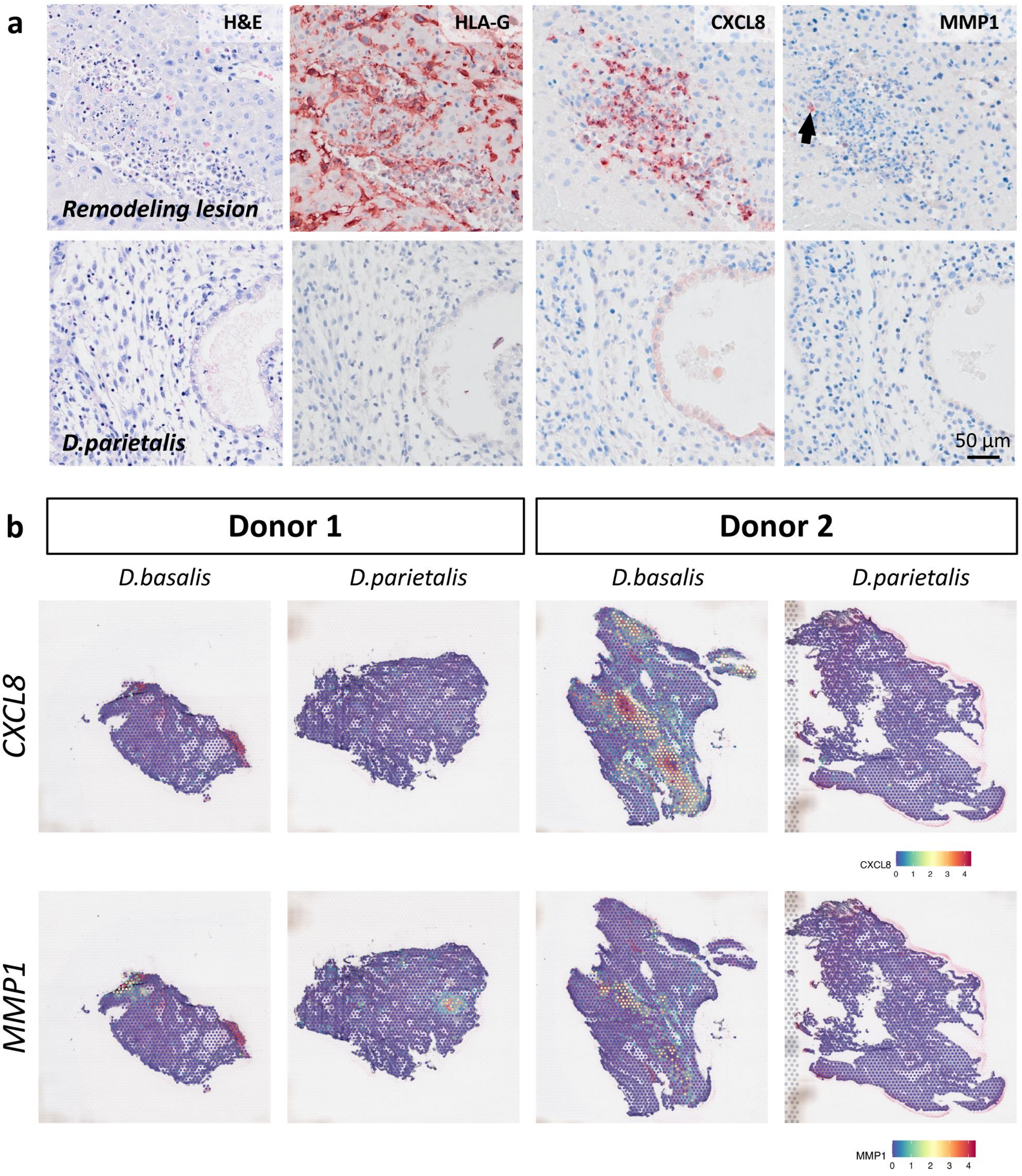

Supplementary Figure S17. **(a) Staining series of selected molecules within a remodeling lesion (first row) and *decidua parietalis* (second row), respectively (exemplified shown for one donor of n = 5).** Consecutive sections (rows) were stained/immunostained for hematoxylin and eosin (H&E) and HLA-G, CXCL8, and MMP1. Nuclear counterstain with hematoxylin. The stainings reveal tissue morphology and EVT invasion beside strong CXCL8 expression within the remodeling lesions, whereas only few cells express MMP1 (black arrow). (**b**) Spatial expression pattern of *CXCL8* and *MMP1* localized in the spatial transcriptomics data (normalized expression, shades from red to blue encode a high to low value range). *Decidua basalis* and *parietalis* from two donors. D., decidua.
