# Supplementary material for: Remodeling lesions locate at sites of strong extravillous trophoblast invasion and are associated with neutrophil presence in the human first-trimester decidua
Source: Hum Reprod. 2026 Jun 5;41(7):1078–96. doi: 10.1093/humrep/deag078 (PMC13334918; doi:10.1093/humrep/deag078)
Supplement: deag078_Supplementary_Figure_S18 [file deag078_supplementary_figure_s18.pdf]

Human Reproduction, 2026, 1–1
https://doi.org/10.1093/humrep/deag078
Supplementary Data

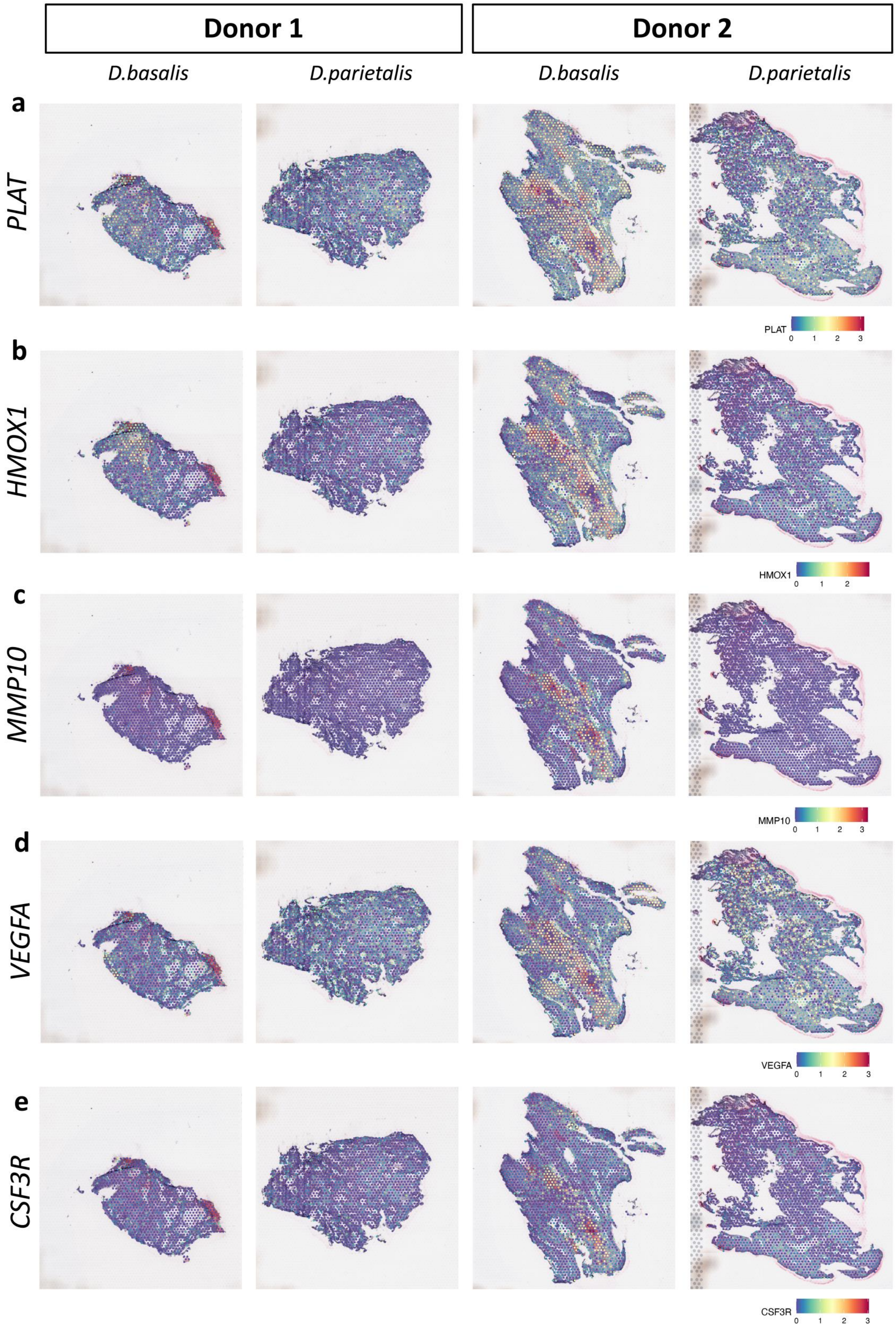

Supplementary Figure S18. The spatial gene expression pattern of selected remodeling lesion markers—(a) *PLAT*, (b) *HMOX1*, (c) *MMP10*, (d) *VEGFA*, and (e) *CSF3R*—localized in the spatial transcriptomics data (normalized expression, shades from red to blue encode a high to low value range). *Decidua basalis* and *parietalis* from two donors. D., decidua.
