# Supplementary material for: Remodeling lesions locate at sites of strong extravillous trophoblast invasion and are associated with neutrophil presence in the human first-trimester decidua
Source: Hum Reprod. 2026 Jun 5;41(7):1078–96. doi: 10.1093/humrep/deag078 (PMC13334918; doi:10.1093/humrep/deag078)
Supplement: deag078_Supplementary_Table_S1 [file deag078_supplementary_table_s1.pdf]

Human Reproduction, 2026, 1–1
https://doi.org/10.1093/humrep/deag078
Supplementary Data

human reproduction
OXFORD

Supplementary Table S1. Characteristics of the donor cohort used in this study.

Parameter, n = 23		
Maternal age (years)	29 [25.5–32.5]	Median [Q1–Q3]
BMI	21.6 [20.7–22.85]	Median [Q1–Q3]
Gestational age (days)	51 [49–54]	Median [Q1–Q3]
Smoker	No = 9; yes = 6	Yes/No; unknown = 8
