# Supplementary material for: Remodeling lesions locate at sites of strong extravillous trophoblast invasion and are associated with neutrophil presence in the human first-trimester decidua
Source: Hum Reprod. 2026 Jun 5;41(7):1078–96. doi: 10.1093/humrep/deag078 (PMC13334918; doi:10.1093/humrep/deag078)
Supplement: deag078_Supplementary_Table_S2 [file deag078_supplementary_table_s2.pdf]

Human Reproduction, 2026, 1–1
https://doi.org/10.1093/humrep/deag078
Supplementary Data

human reproduction
OXFORD

Supplementary Table S2. Antibodies used in this study.

	Antigen	Company	Clone	Cat. No.	Source	Stock concentration	AGR	Dilution		
								IHC FFPE	IF FFPE	IHC cryo
Primary antibodies	CD66b	BD Pharmingen	G10F5	555723	Mouse	0.5 mg/ml	pH 9	1:500	1:200	
	Fibrin	Merck	59D8	MABS2155	Mouse	0.77 mg/ml	pH 9	1:500		
	CD235a	ThermoFisher Scientific	JC159	MA5-44034	Mouse	1 mg/ml	pH 6	1:5000		
	CD31	Abcam	EPR3094	ab76533	Rabbit	0.29 mg/ml	pH 6	1:100		
	HLA-G	BD Pharmingen	4H84	557577	Mouse	0.5 mg/ml	pH 6	1:1000		
	CD3	Dako/Agilent	A0452	A045201-2	Rabbit	0.4 mg/ml	pH 9	1:400	1:100	
	CD8	Dako/Agilent	C8/144B	AM710301-2	Mouse	0.16 mg/ml	pH 9	1:100	1:50	
	CD14	Proteintech	2C1D9	60253-1-Ig	Mouse	1 mg/ml	pH 9	1:2000	1:1000	
	CD163	DB Biotech	K20-T	DB045-01	Rabbit	2 mg/ml	pH 9	1:100	1:50	
	CD56	Dako/Agilent	123C3	M7304	Mouse	0.34 mg/ml	pH 9	1:400		
	CD11c	Abcam	EP1347Y	ab52632	Rabbit	0.11 mg/ml	pH 9	1:500		
	Keratin 7	ThermoFisher Scientific	OV-TL 12/30	MS-1352-P	Mouse	0.2 mg/ml	na			1:5000
	HLA-G	Exbio	MEM-G/9	11-292-C100	Mouse	1 mg/ml	na			1:2000
	CD34	Dako/Agilent	QBEnd 10	M7165	Mouse	0.01 mg/ml	na			1:500
	CD56	Dako/Agilent	MOC-1	M7074	Mouse	0.17 mg/ml	na			1:100
	CXCL8/IL-8	Proteintech	pc	27095-1-AP	Rabbit	0.5 mg/ml	pH 9		1:300	
	MMP1	Proteintech	pc	10371-2-AP	Rabbit	0.7 mg/ml	pH 9		1:600	
	CD14 conj647	Cell signalling	D7A2T	33676	Rabbit	0.4 mg/ml	pH 9		1:100	
	Rabbit Ig Fraction (negative control)	Dako/Agilent	Ig fraction	X0903	Rabbit	20 mg/ml	pH 6/pH 9	*	*	*
	Mouse IgG1 (negative control)	Dako/Agilent	DAK-GO1	X0931	Mouse	0.1 mg/ml	pH 6/pH 9	*	*	*
Secondary antibodies	goat anti mouse CY3	ThermoFisher Scientific		A32727	Goat	2 mg/ml	na		1:200	
	goat anti rabbit CY5	ThermoFisher Scientific		A21070	Goat	2 mg/ml	na		1:200	

AGR, antigen retrieval; IHC, immunohistochemistry; IF, immunofluorescence; FFPE, formalin-fixed paraffin-embedded; Cat. No., catalog number; Ig, Immunglobulin; pc, polyclonal; *, diluted in antibody diluent in concentrations corresponding to the primary antibodies; na, not applicable.
