# Supplementary material for: Remodeling lesions locate at sites of strong extravillous trophoblast invasion and are associated with neutrophil presence in the human first-trimester decidua
Source: Hum Reprod. 2026 Jun 5;41(7):1078–96. doi: 10.1093/humrep/deag078 (PMC13334918; doi:10.1093/humrep/deag078)
Supplement: deag078_Supplementary_Table_S3 [file deag078_supplementary_table_s3.pdf]

Human Reproduction, 2026, 1–1
https://doi.org/10.1093/humrep/deag078
Supplementary Data

human reproduction

OXFORD

Supplementary Table S3. Differences in immune cell populations, HLA-G+ EVTs and erythrocytes, assessed using Friedman's non-parametric test, followed by the Durbin-Conover test for *post-hoc* pairwise comparisons with Holm-adjusted *P*-values (after significant Friedman test with $P < 0.001$).

Group 1	Group 2	HLA-G+ *Padj*	CD56+ *Padj*	CD66b+ *Padj*	CD3+CD8- *Padj*	CD3+CD8+ *Padj*	CD14+ CD163+ *Padj*	CD14+ CD163- *Padj*	Erythrocyt-es *Padj*
Zona compacta	***Zona spongiosa***	0.40465081	2.44E–05	0.01354796	8.16E–08	0.05126146	1.59E–17	0.12633424	3.58E–05
Zona compacta	Weak invasion	1.48E–23	0.57957736	0.09634143	0.19367602	1.63E–05	0.03102185	0.06520104	0.00560334
Zona compacta	Strong invasion	4.62E–39	0.00420345	5.45E–15	0.00250115	0.01503113	6.00E–13	0.25097594	1.15E–18
Zona compacta	Remodeling lesion	1.09E–50	3.19E–08	4.07E–22	4.54E–11	2.78E–08	5.93E–28	3.02E–08	9.52E–24
Zona spongiosa	**Weak invasion**	5.04E–25	3.02E–06	0.36488718	2.96E–05	0.00933733	1.36E–12	0.00013858	0.13477642
Zona spongiosa	Strong invasion	3.68E–40	0.29759087	3.46E–09	0.03225222	4.55E–05	0.03102185	0.60741484	2.64E–09
Zona spongiosa	Remodeling lesion	1.59E–51	0.29759087	2.00E–16	0.19367602	3.71E–12	8.79E–07	0.00013858	1.50E–14
**Weak invasion**	**Strong invasion**	6.22E–15	0.00077687	6.09E–11	0.09245695	1.18E–10	3.26E–08	0.00066742	2.75E–12
Weak invasion	Remodeling lesion	1.56E–32	3.19E–09	3.22E–18	2.89E–08	2.50E–18	1.62E–23	3.68E–13	1.41E–17
**Strong invasion**	**Remodeling lesion**	6.22E–15	0.00804185	0.00181836	0.00018898	0.00106232	5.49E–11	2.08E–05	0.01953582
